# A general framework for updating belief distributions

**DOI:** 10.1111/rssb.12158

**Published:** 2016-02-23

**Authors:** P. G. Bissiri, C. C. Holmes, S. G. Walker

**Affiliations:** ^1^ University of Milano‐BicoccaItaly; ^2^ University of OxfordUK; ^3^ University of Texas at AustinUSA

**Keywords:** Decision theory, General Bayesian updating, Generalized estimating equations, Gibbs posteriors, Information, Loss function, Maximum entropy, Provably approximately correct Bayes methods, Self‐information loss function

## Abstract

We propose a framework for general Bayesian inference. We argue that a valid update of a prior belief distribution to a posterior can be made for parameters which are connected to observations through a loss function rather than the traditional likelihood function, which is recovered as a special case. Modern application areas make it increasingly challenging for Bayesians to attempt to model the true data‐generating mechanism. For instance, when the object of interest is low dimensional, such as a mean or median, it is cumbersome to have to achieve this via a complete model for the whole data distribution. More importantly, there are settings where the parameter of interest does not directly index a family of density functions and thus the Bayesian approach to learning about such parameters is currently regarded as problematic. Our framework uses loss functions to connect information in the data to functionals of interest. The updating of beliefs then follows from a decision theoretic approach involving cumulative loss functions. Importantly, the procedure coincides with Bayesian updating when a true likelihood is known yet provides coherent subjective inference in much more general settings. Connections to other inference frameworks are highlighted.

## 1 Introduction

Data sets are increasing in size and modelling environments are becoming more complex. This presents opportunities for Bayesian statistics but also major challenges, perhaps the greatest of which is the requirement to define the true sampling distribution, or likelihood, for the data generator $f_{0}\left(x\right)$, regardless of the study objective. Even if the task is inference for a low dimensional parameter, Bayesian analysis is required to model the complete data distribution and, moreover, to assume that the model is ‘true’.

In this paper we present a coherent procedure for general Bayesian inference which is based on the updating of a prior belief distribution to a posterior when the parameter of interest is connected to observations via a loss function. Briefly here, and in the simplest scenario, suppose that interest is in the *θ* minimizing the expected loss

(1) $$L\left(\theta \right)=\int l\left(\theta ,x\right)dF_{0}\left(x\right),$$

for some loss function *l*(*θ*,*x*), e.g. *l*(*θ*,*x*)=|*θ*−*x*| for estimating a median, where $F_{0}\left(x\right)$ is the unknown distribution function from which independent and identically distributed observations arise. If *π*(*θ*) represents prior beliefs about this *θ*, and *x* is observed from $F_{0}$, then we argue that a valid and coherent update of *π*(·) is to the posterior *π*(·|*x*), where

(2) π(θ|x)∝exp{−l(θ,x)}π(θ).

The argument for this is given later in the paper and to some extent relies on the idea that an update of beliefs *must* exist. For we have a well‐defined parameter of interest *θ*, an initial belief distribution about the location of the parameter, *π*(*θ*), and gain further independent information about *θ* via *x* coming from $F_{0}\left(x\right)$. To update, it is clear for some function *ψ* that we must have

$$\pi \left(\theta |x\right)=\psi \left\{l(\theta ,x),\pi (\theta )\right\}\;\;.$$

That the form for *ψ* is expression (2) is detailed later and a coherence property plays a key role:

(3) $$\psi \left[l\left(\theta ,x_{2}\right),\psi \left\{l\left(\theta ,x_{1}\right),\pi \left(\theta \right)\right\}\right]\equiv \psi \left\{l\left(\theta ,x_{1}\right)+l\left(\theta ,x_{2}\right),\pi \left(\theta \right)\right\}.$$

This ensures that we end up with $\pi (\theta |x_{1},x_{2})$ as the same object whether we update with $\left(x_{1},x_{2}\right)$ together or $\left\{\left(x_{1}\right),\left(x_{2}\right)\right\}$ one after the other.

A special case is when it is known that $F_{0}\left(x\right)=F\left(x;\theta_{0}\right)$ for some parametric family of distributions *F*(·;*θ*), with corresponding density function *f*(·;*θ*), and *l*(*θ*,*x*)=− log {*f*(*x*;*θ*)}. For minimizing *L*(*θ*) here yields $\theta_{0}$ and the update (2) is the usual Bayesian update. It is important to note that the general Bayesian update using loss functions should not to be seen as an approximation to anything; rather, it is targeting the parameter of interest, employing the necessary loss function with a valid coherent update of beliefs.

Classical inference based on the likelihood function can be regarded as using the ‘negative log‐likelihood function’ as a loss function; for example, in the case of independent and identically distributed observations, we can regard

$$l\left(\theta ;x_{1},\ldots ,x_{n}\right)=-\sum_{i=1}^{n}\log\left\{f\left(x_{i}|\theta \right)\right\}$$

as a loss function connecting data $\left(x_{i}\right)$ with a parameter *θ* indexing the family of density functions *f*(*x*|*θ*). And, in this setting, we do not even need to assume the correctness of the model; we are merely expressing interest in the parameter $\theta_{0}$ minimizing

$$-\int \log\left\{f\left(x;\theta \right)\right\}dF_{0}\left(x\right)$$

which is the parameter minimizing the Kullback–Leibler divergence between the unknown $f_{0}\left(\cdot \right)$ and the family *f*(·;*θ*).

### 1.1 The idea

Here we provide further elaboration on the outline of the idea given previously. Let *θ* denote a parameter or functional of interest, e.g. the mean or median of a population $F_{0}\left(x\right)$, and let *x* denote an observation from $F_{0}\left(x\right)$, with $F_{0}$ unknown. We are interested in a formal way to update prior beliefs *π*(*θ*) to posterior beliefs *π*(*θ*|*x*) given *x*.

Bayesian inference proceeds through knowledge of a complete and true model for $f_{0}\left(x\right)$. This is often parameterized via a sampling distribution *f*(*x*;*θ*) and a prior *π*(*θ*), and defines the marginal likelihood

m(x)=∫f(x;θ)π(θ)dθ.

Then (see for example Bernardo and Smith (1994)), inference for *θ* can occur via Bayes theorem

π(θ|x)=f(x;θ)π(θ)/m(x).

However, the statement ‘inference for *θ*’ is meaningless unless the true parametric family *f*(·;*θ*) is known. In this case, following the Savage axioms (Savage, 1954), the Bayesian update can be shown to be the rational way to proceed. However, $f_{0}\left(x\right)$ may be unknown and, even if *f*(·;*θ*) is correct, *θ* might be ultrahigh dimensional mainly made up of nuisance parameters relative to a low dimensional subset of the parameters of interest. Taken altogether, these points can make the Bayesian approach cumbersome.

We are interested in the rational updating of beliefs under more general and less stringent conditions. To do so we make use of loss functions to connect information in data to parameters of interest. Informally for now, we write such loss functions as *l*(*θ*,*x*), and we shall discuss specific types later in the paper. We shall consider the reporting of subjective beliefs *π*(*θ*|*x*) as an action made under uncertainty and use decision theory to guide the optimal action. See, for example, Hirshleifer and Riley (1992).

To outline the theory, let *ν* denote a probability measure on the space of *θ*. We shall construct a loss function to select an optimal posterior distribution $\hat{\nu }\left(\theta \right)$ given a prior *π*(*θ*) and data *x*. (We use $\hat{\nu }$ to denote optimality rather than an approximation or estimate.) To achieve this we construct a loss function *L*(*ν*;*π*,*x*) on the space of probability measures on *θ*‐space, and then present

$$\hat{\nu }=\arg\underset{\nu }{\text{min}}.5ptL\left(\nu ;\pi ,x\right)$$

as the representation of beliefs about the unknown value of *θ* given the prior information, represented via the belief distribution *π*, and data *x*. As it is widely assumed that data *x* are an independent piece of information to that which gave rise to the prior, it is appropriate to consider an additive, or cumulative, loss function of the form

(4) $$L\left(\nu ;\pi ,x\right)=h_{1}\left(\nu ,x\right)+h_{2}\left(\nu ,\pi \right),$$

where $h_{1}$ and $h_{2}$ are themselves loss functions on probability measures, representing fidelity to data and fidelity to prior respectively. See, for example, Berger (1993) for more about ideas on uses of loss functions within decision theory.

The question is whether we can claim a probability measure selected as the solution to a decision problem, i.e. minimizing a loss function, can be viewed as representing beliefs about a parameter. To answer this, given the aim (1), we would clearly prefer probability measure $\nu_{1}$ to $\nu_{2}$ as representing beliefs if

(5) $$\int \int l\left(\theta ,x\right)dF_{0}\left(x\right)\nu_{1}\left(d\theta \right)⩽\int \int l\left(\theta ,x\right)dF_{0}\left(x\right)\nu_{2}\left(d\theta \right).$$

Indeed, it would be incoherent to select $\nu_{2}$ rather than $\nu_{1}$ when condition (5) holds. Thus the answer is affirmative. Though we are not minimizing or comparing condition (5), since we do not have $F_{0}$, we can substitute the expression

(6) $$L_{0}\left(\nu ;F_{0}\right)=\int \int l\left(\theta ,x\right)dF_{0}\left(x\right)\nu \left(d\theta \right)$$

with the Bayesian finite sample expression of the form (4). We now discuss the choices of $h_{1}$ and $h_{2}$ which give equation (4) as a Bayesian finite sample version of equation (6).

Under this approach the analyst needs to specify $h_{1}$ and $h_{2}$ in such a way that they proceed in an optimal, rational and coherent manner. Somewhat remarkably, as proved in the on‐line supplementary material, for coherent inference (3), $h_{2}$ must be the Kullback–Leibler divergence (Kullback and Leibler, 1951), and given by

$$h_{2}\left(\nu ,\pi \right)=d_{\mathrm{KL}}\left(\nu ,\pi \right)=\int \nu \left(d\theta \right)\log\left\{\nu \left(d\theta \right)/\pi \left(d\theta \right)\right\},$$

where, with a slight abuse of notation, we also use *π*(d*θ*) to denote the probability measure version of *π*, i.e. *π*(d*θ*)=*π*(*θ*) d*θ*.

Regarding $h_{1}$, since *ν*(*θ*) is a probability measure representing beliefs about *θ*, the only choice here is to take the loss to data $h_{1}\left(\nu ,x\right)$ as the *expected* loss (see von Neumann and Morgenstern (1944)) of *l*(*θ*,*x*), i.e.

$$h_{1}\left(\nu ,x\right)=\int l\left(\theta ,x\right)\nu \left(d\theta \right),$$

with the particular types of the loss function on the parameter of interest *l*(*θ*,*x*) to be discussed later.

Substituting in $h_{1}$ and $h_{2}$, the cumulative loss function is then given by

(7) $$L\left(\nu ;\pi ,x\right)=\int l\left(\theta ,x\right)\nu \left(d\theta \right)+d_{\mathrm{KL}}\left(\nu ,\pi \right).$$

This then, i.e. equation (7), is our finite sample version of equation (6), and note that equation (7) becomes, under mild regularity conditions, equation (6) as *n*→∞. The solution to equation (7) provides the $\hat{\nu }$ which the statistician believes best minimizes equation (6). This is, according to our approach, done by using the empirical distribution function as a substitute for $F_{0}$ and using a penalty term which prevents the answer from being too far from the prior in a Kullback–Leibler sense; the Kullback–Leibler appearing here for the necessary coherence property of the answer. Of interest, as discussed later on, is the provably approximately correct (PAC) Bayes solution to the problem (Langford, 2005) that finds an approximation which minimizes an upper bound for equation (6); see Section 3.

Surprisingly, but quite easy to show, the minimizer of *L*(*ν*;*π*,*x*) is given by

(8) $$\begin{array}{cc} \hat{\nu }\left(\theta \right)= & \arg\min_{\nu }L\left(\nu ;\pi ,x\right)\\ = & \frac{\exp\{-l(\theta ,x)\}\pi (\theta )}{\int .1pt\exp\{-l(\theta ,x)\}\pi (d\theta )}. \end{array}$$

This can be seen by observing that

$$\int l\left(\theta ,x\right)\nu \left(d\theta \right)+d_{\mathrm{KL}}\left(\nu ,\pi \right)=\int \nu \left(d\theta \right)\log\;\;\left[\frac{\nu (\theta )}{\exp\{-l(\theta ,x)\}\pi (\theta )}\right]\;\;.$$

So equation (8) has the form of a Bayesian update using exponentiated negative loss in place of the likelihood function. We stress again that equation (8) is not an approximation, or pseudoposterior, but rather a valid coherent representation of subjective uncertainty in the minimizer of expression (1). As is usual in decision problems involving the use of loss functions, it is incumbent on the decision maker to ensure that solutions exist. So *l*(*θ*,*x*) needs to be constructed such that

0<∫.3ptexp{−l(θ,x)}π(dθ)<∞.

Whereas the Bayesian approach requires the construction of a probability model for all possible outcomes conditionally on all unknown states of nature, the general Bayesian approach requires the construction of loss functions given the outcomes for only the parameter of interest. This allows the decision maker to concentrate on modelling only those quantities that are important to the task at hand.

### 1.2 On equation (3) implying equation (2)

We shall now go into the details of how equation (3) and some other natural assumptions imply equation (2). We are asking for the unique *ψ* which provides the update for all *Θ*, i.e. is *Θ* invariant? This is a reasonable requirement since how we update should not depend on *Θ*. In fact we show that equation (3) uniquely implies equation (2) for |*Θ*|=3, i.e. the cardinality of *Θ* is 3, and hence the update is the only unique update that applies for all *Θ*. So consider the following assumptions.

Assumption 1 Condition (3) holds true.

Assumption 2 For any set *A*⊂*Θ*,

(9) $$\frac{\psi \{l(\theta ,x),\pi (\theta )\}}{\int_{\;\;\;\;A}\psi \left\{l\left(\theta ,x\right),\pi \left(\theta \right)\right\}d\theta }=\psi \left\{l\left(\theta ,x\right),\pi_{A}\left(\theta \right)\right\},$$

where $\pi_{A}$ is *π* restricted and normalized to *A*, i.e. $\pi_{A}\left(\theta \right)=\pi \left(\theta \right)1\left(\theta \in A\right)/\int_{A}.5pt\pi \left(\theta \right)d\theta$. This condition says that whether we update the prior restricted to the set *A*, or update the prior and then restrict to the set *A*, we obtain the same update.

Assumption 3 Lower evidence (larger loss) for a state should yield smaller posterior probabilities under the same prior. So, if for some *A*⊂*Θ*,* l*(*θ*,*x*)>*l*(*θ*,*y*) for *θ* ∈ *A*⊂*Θ* and *l*(*θ*,*x*)=*l*(*θ*,*y*) for $\theta \in A^{c}$, then

$$\int_{A}\psi \left\{l\left(\theta ,x\right),\pi \left(\theta \right)\right\}d\theta <\int_{A}\psi \left\{l\left(\theta ,y\right),\pi \left(\theta \right)\right\}d\theta .$$

Assumption 4 If *l*(*θ*,*x*)≡constant, then *ψ*{*l*(*θ*,*x*),*π*(*θ*)}=*π*(*θ*), i.e., if the observation provides no information about *θ*, since the loss function is a constant, then the posterior is the same as the prior.

Assumption 5 If  $\tilde{l}\left(\theta ,x\right)=l\left(\theta ,x\right)+c$ for some constant *c*, then

$$\psi \left\{\tilde{l}\left(\theta ,x\right),\pi \left(\theta \right)\right\}=\psi \left\{l\left(\theta ,x\right),\pi \left(\theta \right)\right\}.$$

Theorem 1 If assumptions 1–5 hold, then for |*Θ*|=3 equation (3) uniquely implies equation (2).

The proof is given in Appendix A. It is quite straightfoward to extend the uniqueness argument to all countably infinite *Θ*, which would replace the uniqueness argument for all *Θ*. However, we would need more work to extend separate uniqueness to general *Θ*.

Clearly Sections 1.1 and 1.2 are different derivations of the same result, i.e. the support of update (2).

### 1.3 Connections with related work

There is a large literature on procedures for robustly estimating a parameter of interest by minimizing the cumulative loss

(10) $$L\left(\theta ;x\right)=\sum_{i=1}^{n}.5ptl\left(\theta ,x_{i}\right).$$

This is clearly the finite sample version of

$$L\left(\theta \right)=\int l\left(\theta ,x\right)dF_{0}\left(x\right).$$

Our claim is that equation (7) is the general Bayesian version of equation (10), where interest is on probability measures on *θ*‐space rather than single states *θ*.

Hüber (2009) provided examples of equation (10), where we note that the primary aim is not modelling the data but rather estimating a parameter. This is an advantage when a probability model for the data is too difficult to formulate. We are presenting a general Bayesian extension of this idea. Since we are interested in a belief distribution for *θ* given data, and we have further information provided by *π*, we claim that the appropriate Bayesian version is given by equation (8).

Some of the ideas that are presented in the paper have been considered by Zhang (2006a,b) and Jiang and Tanner (2008). In Zhang (2006a) an estimation procedure, named information risk minimization, also known as a Gibbs posterior, which has the same form as equation (8), is described in section IV of his paper. Zhang then concentrated on the properties of the Gibbs posterior. Further theoretical work was done in Zhang (2006b).

In Jiang and Tanner (2008) a Gibbs posterior was studied in comparison with a true Bayesian posterior where the model is assumed to be misspecified. The claim is that posterior performance of a Bayesian model can be unreliable when misspecified, whereas a Gibbs posterior which targets points of interest can have better performance. The comparison involves variable selection for high dimensional classification problems involving a logit model.

Here we show that solutions of the form (8) are the only coherent, decision theoretic representation of posterior beliefs under model misspecification. We also provide a principled approach to scale the relative information in the data to information in the prior (see Section 3); that was left as an arbitrary free parameter in Zhang (2006a,b) and Jiang and Tanner (2008).

Bissiri and Walker (2010) used equation (7) with Bernoulli observations and found sufficient conditions on *l*(*θ*,*x*) for the sequence of posteriors, based on equation (8), to be consistent. This result for consistency was extended to more general independent and identically distributed observations in Bissiri and Walker (2012a). In Bissiri and Walker (2012b), it was shown starting from the class of *g*‐divergences (Ali and Silvey, 1966), for a coherent sequence of updates, that we need the Kullback–Leibler divergence as the loss between prior *π* and *ν*. In the on‐line supplementary material, we present an updated proof that is simplified and more intuitive to that appearing before now.

A similar construct to *L*(*ν*;*π*,*x*) was provided by Zellner (1988), who presented what is essentially a loss function for the posterior distribution by using ideas of information processing from prior to posterior. The motivation is different and relies on notions of information present in log‐probabilities and log‐likelihoods, which may not be compatible as noted by J. M. Bernardo in the discussion of Zellner (1988). Furthermore, our derivation of the loss function allows a broader interpretation of the elements, which does not require the existence of a probability distribution for the observation; see Section 4.

Concerns that the specification of a complete model for the data‐generating distribution is unachievable date back to de Finetti (1937) and the notion of ‘prevision’. In his work de Finetti considered conditional expectation as the fundamental primitive, or statistic, of interest on which prior beliefs are expressed and updated. Recently other researchers have further developed this approach under the field of Bayesian linear statistics; see Goldstein and Wooff (2007).

There has been increasing awareness of the restrictive assumptions that formal Bayesian analysis entails. Royall and Tsou (2003) described procedures for adjusting likelihood functions when the model is misspecified. More recently, Doucet and Shephard (2012) and Müller (2012) considered formal approaches to pseudo‐Bayesian methods using sandwich estimators to update subjective beliefs, motivated by robustness to model misspecification; see also Hoff and Wakefield (2013). Cooley *et al*. (2009) considered pseudo‐Bayesian approaches with composite likelihoods. More generally there is increasing recognition that formal Bayesian analysis can be restrictive for example through computational issues, such as arise in the area of approximate Bayesian computation (see, for example, Marin *et al*. (2012)).

Several researchers have considered issues with Bayesian updating by using proxy models *f*(*x*;*θ*) (for example, see Key *et al*. (1999)), when $\left(x_{i}\right)$ are known not to arise from *f*(*x*;*θ*) for any value of *θ*, i.e. there is no *θ* conditional on which *x* is from *f*(*x*;*θ*). This is referred to as the *M*‐open case in Bernardo and Smith (1994). One suggested solution is to use methods based on approximations and Key *et al*. (1999) described one such idea using a cross‐validation approach. Although this may be pragmatic it does have some shortcomings. Most serious is that there is little back‐up theory and this has repercussions in that the update suffers from a lack of coherence.

Another approach is to ignore the problem, i.e. to assume that the observations are coming from *f*(*x*;*θ*) even though it is known that they are not. According to Goldstein (1981), ‘there is no obvious meaning for Bayesian analysis in this case’. The disaster of making horribly wrong inference can be protected to some extent by model selection, i.e. postulating a number of models for $f_{0}\left(x\right)$, say $f_{j}\left(x;\theta_{j}\right)$, with corresponding priors $\pi_{j}\left(\theta_{j}\right)$, and model probabilities $p_{j}$, for *j*=1,…,*M*. But, as Key *et al*. (1999) pointed out, how do we construct $\pi_{j}\left(\theta_{j}\right)$ and $p_{j}$ when we know that none of the postulated models are correct? So the Bayesian update breaks down in that nothing has any interpretation.

Finally, and we acknowledge the contribution of the reviewers for pointing this out, we discuss connections with PAC Bayes methods; see, Shawe‐Taylor and Williamson (1997), Langford (2005), Alquier (2008) and McAllester (1998). PAC Bayes is an interesting emerging field in machine learning concerned with techniques for bounding the generalization error (empirical risk) of a Bayesian model. The motivation behind PAC Bayes methods is to find an upper bound for the empirical risk of a probability measure *ν* on a model $L(\nu ;F_{0})$ in equation (6), which is termed generalization error in the PAC Bayes literature. Given observation *x* and prior *π*, the upper bound will be written as *U*(*ν*;*x*,*π*), i.e. for all *ν*

$$L\left(\nu ;F_{0}\right)⩽U\left(\nu ;x,\pi \right).$$

See Catoni (2003) where the form of *U* is provided. Then it can be shown that an upper bound *U*(*ν*;*x*,*π*) is provided by equation (8). The PAC Bayes approach is complementary to our work. The motivation and construction are very different. We are interested in a framework for the rational updating of beliefs, rather than seeking bounds on the empirical risk of a probability measure on models. The minimizer of an upper bound is interesting but does not justify using $\hat{\nu }$ as an update of a belief distribution for Bayesian style inference, and hence whether $\hat{\nu }$ forms a coherent sequence of belief distributions is not discussed in the PAC Bayes formulation of *U*; the requirement of coherence is central to Bayesian style learning. Moreover the scaling of the loss to data $h_{1}$ to the loss to prior $h_{2}$ enters as a constant in the margin of the error bound in PAC Bayes methods, whereas here it has explicit meaning in the relative weight of information provided by the two sources, prior and data (see Section 3).

The general Bayesian approach also coincides with the prediction‐motivated approach of Cesa‐Bianchi and Lugosi (2006) and is known as aggregation with exponential weight, which does not rely on stochastic information; see also our Section 4.1.

This said, there are clear synergies and the operational characteristics of PAC Bayes methods are similar; they must be since we gather the same answer. However, the motivation and consequences are different. Moreover, as we shall see later, the derivation here provides insights into the necessary calibration of loss functions $h_{1}$ and $h_{2}$.

### 1.4 Layout of the paper

The layout of the remainder of the paper is as follows. In Section 2 we discuss types of loss function. When the self‐information loss function is used then the update is the traditional Bayes update. With other loss functions there is a calibration issue between the two styles of loss function used, i.e. the loss to the data and the loss to the prior. This calibration problem is discussed and potential solutions provided in various ways in Section 3. In Section 4 we discuss forms of information other than the usual data arising from some unknown distribution function. This includes non‐stochastic information and also partial information. Section 5 provides some numerical illustrations including inference based on partial information and a clustering problem. Section 6 concludes with a discussion on various points.

The programs that were used to analyse the data can be obtained from

http://wileyonlinelibrary.com/journal/rss-datasets

## 2 Types of loss function

In this section we shall consider the form of $h_{1}$ in equation (4) that connects information in the data to the value of the unknown *θ*. We shall consider three broad situations: first, when the analyst believes that they know the complete family of distributions from which the $\left(x_{i}\right)$ arose, the so called *M*‐closed scenario; second, when $f_{0}\left(x\right)$ is unknown but where a complete likelihood *f*(*x*;*θ*) is being used as a proxy model; finally, when there is no sampling distribution or proxy model for *x* and the parameter of interest is connected to *x* via a loss function *l*(*θ*,*x*).

### 2.1 M‐closed case and self‐information loss

When the analyst knows the family from which $\left(x_{i}\right)$ arose, the so‐called *M*‐closed view, then the Bayesian approach to learning is fully justified, well known and widely used as a statistical approach to inference; Bernardo and Smith (1994) is comprehensive. To see how Bayes arises in our framework, we would need to construct a loss function for *l*(*θ*,*x*) with the knowledge that *x* came from *f*(*x*;*θ*). It is well known that the appropriate and sole loss function in this case is the self‐information, or logarithmic loss function, given by

l(θ,x)=−log{f(x;θ)}.

Indeed, the cumulative loss version of this is the log‐likelihood function. See Bernardo (1979) and Merhav and Feder (1998) for more on the self‐information loss function. This amounts to the use of proper scoring rules when the parametric family *f*(*x*;*θ*) is known, and under which our approach coincides with the Bayesian updating rule.

### 2.2 M‐open case and the use of proxy models

Issues with the Bayesian rule arise when the form of *f*(*x*;*θ*) is not known; for example, see Key *et al*. (1999). Equivalently, there is no *θ* conditional on which *x* is from *f*(*x*;*θ*); more bluntly, there is no connection between any *x* and any *θ* via *f*(*x*;*θ*). This is referred to as the *M*‐open case in Bernardo and Smith (1994). In many situations, the correct sampling density $f_{0}\left(x\right)$ is unknown or unavailable or too complex to work with.

Under a general Bayesian approach we may proceed by considering $\theta_{0}$, the value of *θ* that minimizes the Kullback–Leibler divergence between a proxy model *f*(*x*;*θ*) and the true density function $f_{0}\left(x\right)$, i.e. $\theta_{0}$ minimizes

$$d_{\mathrm{KL}}\left\{f_{0}\left(\cdot \right),f\left(\cdot ;\theta \right)\right\}=\int f_{0}\left(x\right).5pt\log\left\{f_{0}\left(x\right)/f\left(x;\theta \right)\right\}dx.$$

Then prior beliefs *π*(*θ*) will be expressed on this unknown value. It is possible to learn about this $\theta_{0}$ since an infinite collection of $\left(x_{i}\right)$ yields $\theta_{0}$. Then we would wish the sequence of *ν*(*θ*) to accumulate about $\theta_{0}$. The appropriate loss function in this case is still *l*(*θ*,*x*)=− log {*f*(*x*;*θ*)}. The standardized cumulative loss based on a sequence of observations ${\left(x_{i}\right)}_{i=1}^{n}$ is given by $-n^{-1}\Sigma_{i=1}^{n}.5pt\log\left\{f\left(x_{i};\theta \right)\right\}\to -\int \log\left\{f\left(x;\theta \right)\right\}dF_{0}\left(x\right)$ almost surely for all *θ*, which is minimized by $\theta_{0}$.

So although the Bayesian approach has foundational issues to deal with whether the *M*‐open or *M*‐closed view holds, for the approach here it is irrelevant. If we adopt $\theta_{0}$ as the parameter value taking the family closest to $f_{0}\left(\cdot \right)$ then we do not need to worry if we are in the *M*‐open or *M*‐closed scenario, since if *f*(·;*θ*) is the true family then obviously $\theta_{0}$ reverts to the true parameter value. This point is crucial, since for the Bayesian being in the *M*‐open or *M*‐closed state forces us to adopt different inference approaches; see Bernardo and Smith (1994). Moreover our approach supports the use of the relevant partial information in the data for updating beliefs on the parameter of interest, an example of which is shown in Section 5.1. This can be especially important when the data are high dimensional. Such updates have no formal justification from a Bayesian perspective.

### 2.3 Parameter minimizing a loss function

In the most general scenario the parameter of interest minimizes a loss function of the type (1). In the classical literature, this type of estimation problem is in the area of *robust statistics* and specific loss functions can be found in the literature, pertaining to *M*‐estimation and estimating equations. See, for example, Hüber (2009).

An important class of loss functions is provided by the *M*‐estimators for a location parameter; Hüber (1964). So, rather than using the loss function $-\log\{f\left(x_{i};\theta \right)\}$, a $\rho (x_{i};\theta )$ is used in an attempt to obtain robust estimation, rather than the traditional maximum likelihood estimator, which can be suspect if the model is incorrect. This idea has been generalized to the class of estimating equations, whereby the estimate of *θ* is obtained by minimizing

$$\sum_{i=1}^{n}.3pt\rho \left(x_{i};\theta \right).$$

Our approach, which mirrors this classical robust procedure, would use the loss function

$$L\left(\nu ;x_{1},\ldots ,x_{n},\pi \right)=\int \sum_{i=1}^{n}.1pt\rho \left(x_{i};\theta \right)\nu \left(d\theta \right)+d_{\mathrm{KL}}\left(\nu ,\pi \right)$$

with solution provided by

$$\hat{\nu }\left(d\theta \right)\propto \exp\;\{\;\;-\sum_{i=1}^{n}.1pt\rho \left(x_{i};\theta \right)\}\pi \left(d\theta \right).$$

The $\theta_{0}$ of interest is implicitly assumed to be the limit of the sequence of minimizers of the cumulative losses. This would be the minimizer of $\int .1pt\rho \left(x;\theta \right)dF_{0}\left(x\right)$ and hence the prior beliefs are being expressed about this unknown value. Then the loss function *l*(*θ*,*x*)=*ρ*(*x*;*θ*) is ensuring that the updates are indeed ‘moving towards’ $\theta_{0}$. To complete the picture, it would have been that the decision maker would be happy to make a decision given the minimizer of $\int .1pt\rho \left(x;\theta \right)dF_{0}\left(x\right)$.

## 3 Calibration of relative losses

This section deals with the important aspect of specifying the relative information in the data to the information in the prior in general settings. In the *M*‐closed and *M*‐open, including partial information, settings the use of the self‐information loss *l*(*θ*,*x*)=− log {*f*(*x*;*θ*)} results in a fully specified form for equation (8). However, in the setting of Section 2.3 there is an issue about the scale of the loss function $h_{1}$ which is a consequence of the apparent arbitrariness in the weight of $h_{1}\left(\nu ,x\right)$ relative to $h_{2}\left(\nu ,\pi \right)$, in that we are free to multiply either by an arbitrary factor. So, equivalently, we are interested in a loss function *w* *l*(*θ*,*x*) for some *w*>0. The question is how to select *w*, noting that *w* controls the relative weight of loss to data to loss to prior.

Of course, such an issue does not arise in general in the classical literature on *parameter* estimation since there is typically no combining with different styles of loss function. A notable exception is the class of regularized regressions, such as the lasso, where one minimizes

$$L\left(\beta \right)=w\sum_{i=1}^{n}l\left(\beta ,y_{i},x_{i}\right)+\left|\beta \right|.$$

Note the substantial difference in that this loss is for a parameter, whereas the losses that we consider are for a measure.

The calibration of different types of loss is not a unique problem to us or to the lasso. It arises in many applied contexts; possibly the most well known are in health economics where losses pertaining to costs need to be balanced against losses pertaining to health benefits.

The most common ideas for assigning *w* in the Gibbs posteriors and PAC Bayes literature typically involve cross‐validation and subjective choices. As mentioned above, in PAC Bayes methods the weighting *w* is a constant that enters the margin of the error bound. Here we discuss some ideas in the context of a general Bayesian update intended to help the analyst. We do not claim to be exhaustive in the approaches, or to be prescriptive in advocating one approach over another. Our intention is to provide tools and suggestions for elicitation of the relative loss to data to loss to prior.

### 3.1 Annealing

In the literature on Gibbs posteriors, the weighting parameter is labelled as a ‘temperature’ and selected subjectively. There are clear connections here with the use of ‘power priors’ (Ibrahim and Chen, 2000) where

$$\nu \left(d\theta \right)\propto \prod_{i=1}^{n}f{\left(x_{i};\theta \right)}^{w}\pi \left(d\theta \right).$$

Such an idea has also been discussed in Walker and Hjort (2001). It is evident what *w* achieves; if 0<*w*<1 then the loss to prior is given more prominence than in the Bayesian update and the data will be less influential. In the extreme case when *w*=0 we retain the prior throughout. In contrast, when *w*>1 the loss − log {*f*(*x*;*θ*)} is given more prominence than in the Bayesian update and in the extreme case when *w* is very large the *ν* is accumulating about the maximum likelihood estimator for the model, i.e.

$$\nu \left(d\theta \right)\approx \delta_{\hat{\theta }}\left(d\theta \right),$$

where $\hat{\theta }$ maximizes $\Pi_{i=1}^{n}f\left(x_{i};\theta \right)$.

### 3.2 Unit information loss

Here we discuss a procedure for default subjective assignment based on a prior evaluation of the expected value of *l*(*θ*,*x*). The idea originates from work in the specification of reference priors and ‘objective Bayes’ methods; see for example Kass and Wasserman (1996).

To begin it helps to ensure that both losses are non‐negative for all *θ*. Hence we write the prior loss function with an additional term $\log\{\pi \left(\hat{\theta }\right)\}$, which is a constant, and where $\hat{\theta }$ maximizes *π*(*θ*), so that the cumulative loss becomes

$$L\left(\nu ;x,\pi \right)=\int \left[wl\left(\theta ,x\right)+\log\left\{\pi \left(\hat{\theta }\right)/\pi \left(\theta \right)\right\}\right]\nu \left(d\theta \right)+\int \nu \left(d\theta \right).2pt\log\left\{\nu \left(\theta \right)\right\}.$$

and we would additionally standardize *l*(*θ*,*x*) such that $\min_{\theta }.3ptl\left(\theta ,x\right)=0$ for any *x*. Hence, we can regard

$$L\left(\theta ;x,\pi \right)=wl\left(\theta ,x\right)+\log\{\pi \left(\hat{\theta }\right)/\pi \left(\theta \right)\}$$

as a loss function for *θ* with information provided by *x* and *π*. So, assuming that *l*(*θ*,*x*)>0, we want to calibrate the two loss functions given by

wl(θ,x)

and

$$\log\{\pi \left(\hat{\theta }\right)/\pi \left(\theta \right)\}.$$

These are two loss functions for *θ* and to adhere with the notion that, before we have any data, there is a single piece of information, we can calibrate the two losses by making the joint expected losses, taken over *θ* and *x*, to match, i.e. whether someone takes a *θ* and is penalized by the loss

$$\log\{\pi \left(\hat{\theta }\right)/\pi \left(\theta \right)\},$$

or takes a (*θ*,*x*) and is penalized by the loss *w* *l*(*θ*,*x*), at the outset, the expected losses should match. They are confronted by two choices of loss with one piece of information and thus the losses can be calibrated by ensuring that their expected losses coincide. The connection between expected information and expected loss can be found in Bernardo (1979).

Thus *w* can be set by ensuring that

$$wE_{\theta ,\;x}\left\{l\left(\theta ,x\right)\right\}=E_{\theta }\left[\log\left\{\pi \left(\hat{\theta }\right)/\pi \left(\theta \right)\right\}\right].$$

Here *E* is with respect to a joint belief distribution in *x* and *θ*; say *m*(*x*,*θ*), the marginal for *θ* of which is *π*(*θ*). So

(11) $$w=\frac{\int \log\left\{\pi \left(\hat{\theta }\right)/\pi \left(\theta \right)\right\}\pi \left(d\theta \right)}{\int \int l(\theta ,x)m(d\theta ,x\_\_)}.$$

Let us consider an example, where $l\left(\theta ,x\right)={\left(\theta -x\right)}^{2}$ with $\pi \left(\theta \right)=N\left(\theta |0,\tau^{2}\right)$ with *m*(*x*|*θ*) being any density with mean *θ* and variance $\sigma^{2}$. Then we can evaluate

$$\int \log\left\{\pi \left(\hat{\theta }\right)/\pi \left(\theta \right)\right\}\pi \left(d\theta \right)=\frac{1}{2}$$

and

$$\int \int {\left(\theta -x\right)}^{2}m\left(dx,d\theta \right)=\sigma^{2},$$

so $w=\frac{1}{2}\sigma^{-2}.$ Hence, this calibration idea yields the ‘correct’ value of $\frac{1}{2}\sigma^{-2}$ in this case. This construction requires the user specification of a joint density *m*(d*x*,d*θ*) which in some circumstances may prove difficult. Here we propose an empirical expression for this.

Now $x_{-i}=\left(x_{1},\ldots ,x_{i-1},x_{i+1},\ldots ,x_{n}\right)$ should predict $x_{i}$ and the best *θ*‐value to achieve this would minimize

$$\sum_{j\neq i}.3ptl\left(\theta ,x_{j}\right).$$

Denote the minimizer as ${\hat{\theta }}_{-i}$. Then we would empirically estimate the denominator of equation (11) by

$$E\left\{l\left(\theta ,x\right)\right\}=n^{-1}\sum_{i=1}^{n}.5ptl\left({\hat{\theta }}_{-i},x_{i}\right).$$

To see this more easily, we relate it to a standard Bayesian cross‐validation idea. So assume that we wish to estimate

(12) E[log{f(x|θ)}]

empirically. Given $x_{-i}$ we would predict $x_{i}$ by using the plug‐in density $f(x_{i}|{\hat{\theta }}_{-i})$, where ${\hat{\theta }}_{-i}$ maximizes $\Pi_{j\neq i}f\left(x_{j}|\theta \right)$. Hence, we would estimate expectation (12) via

$$n^{-1}\sum_{i=1}^{n}\log\left\{f\left(x_{i}|{\hat{\theta }}_{-i}\right)\right\}$$

based on the idea that $\left({\hat{\theta }}_{-i},x_{i}\right)$ represent an empirical sample from *m*(*θ*,*x*).

If we illustrate this on a toy example, for which $l\left(\theta ,x\right)={\left(x-\theta \right)}^{2}$ and *π*(*θ*) is normal with zero mean and variance 1/*τ*, then it is easy to show that

$$w=\frac{1}{2}\frac{n}{\sum_{i=1}^{n}{\left(x_{i}-{\overset{¯}{x}}_{-i}\right)}^{2}}$$

which asymptotically becomes $\frac{1}{2}\sigma^{-2}$, with $\sigma^{2}$ the variance of the data.

It is interesting to note in the above that if it is thought that the appropriate choice for *π*(*θ*) is flat, which is possible if the *θ*‐space is bounded, then clearly we have $\log\{\pi \left(\hat{\theta }\right)/\pi \left(\theta \right)\}=0$. Thus, to be coherent, we would equally believe that ∫ *ptl*(*θ*,*x*) *m*(d*x*|*θ*) does not depend on *θ*, where *m*(·|*θ*) is a belief distribution for *x* given *θ*. This is a condition which would be difficult to justify, as it would then be also for the uniform prior for *θ*. If one is used, then we only recommend that the value of *w* is not assigned in the above way.

### 3.3 Hierarchical loss

Another way to proceed is to extend the loss function to include *w* as an unknown parameter. Standard ideas here would suggest that we take

L(θ,w;x,π)=wl(θ,x)+ξl(w)−log{π(θ,w)}

for some *ξ*>0. We would appear to be making no progress since we now have a *ξ* to assign. However, this is akin to the hierarchical Bayesian model where uncertainty is propagated via hyperprior distributions to robustify the ultimate prior choice at some level. Hence, the allocation of a *ξ* would not be as crucial as the assignment of a *w*.

For example, as *w* is a scale parameter on loss to data, taking *l*(*w*)= log (*w*) the solution is given by

$$\hat{\nu }\left(\theta ,w|x,\pi \right)\propto w^{\xi }.5pt\exp\left\{-wl\left(\theta ,x\right)\right\}\pi \left(\theta ,w\right)$$

and given that $w^{\xi }$ can be absorbed in the prior *π* it is reasonable to assess *ξ* subjectively, i.e. it seems unreasonable to accept that *π* can be chosen subjectively but that *ξ* cannot.

### 3.4 Operational characteristics and subjective calibration

The idea here is to set *w* so that the posterior quantiles are calibrated at some level of error to frequentist confidence intervals based on the estimation of *θ* via minimizing the loss

$$\sum_{i=1}^{n}l\left(\theta ,x_{i}\right).$$

So, if $C_{\alpha }\left(w,x_{1},\ldots ,x_{n}\right)$ is the 100(1−*α*)% level confidence interval for *θ*, then we could select the *w* such that the posterior distribution of *θ*, with parameter *w*, is such that

$$P\{\theta \in C_{\alpha }\left(w,x_{1},\ldots ,x_{n}\right)|x_{1},\ldots ,x_{n}\}=1-\alpha .$$

See, for example, Datta and Sweeting (2005) for references to probability matching priors and posteriors, and Cooley *et al*. (2009) for ideas in pseudo‐Bayesian approaches with composite likelihoods.

More generally we can consider the subjective setting of *w* where knowledge of the frequentist sampling statistic of $\Sigma_{i=1}^{n}.5ptl\left(\theta ,x_{i}\right)$ can assist. To begin note that *w* is explicitly related to the Bayes factor quantifying the posterior‐to‐prior odds,

$$\log\;\left\{\frac{\pi (\theta |x)}{\pi (\theta^{\prime }|x)}/\frac{\pi (\theta )}{\pi (\theta^{\prime })}\right\}=-w\left\{l\left(\theta ,x\right)-l\left(\theta^{\prime },x\right)\right\}$$

where $w\{l\left(\theta ,x\right)-l\left(\theta^{\prime },x\right)\}$ measures the update in beliefs in favour of *θ* from $\theta^{\prime }$ on observing *x*. Clearly the larger the difference $l\left(\theta ,x\right)-l\left(\theta^{\prime },x\right)$ is the greater the relative evidence in favour of *θ*, with *w* determining the scale for unit change. It is interesting to note that, should the Bayes factor be known for any three points $\left\{\theta ,\theta^{\prime },x\right\}$ in the joint parameter sample space, $\Omega_{\theta^{2}}\times \Omega_{X^{n}}$, then *w* would be fixed. The idea here is that the analyst is free to contemplate any specific values $\left\{\theta ,\theta^{\prime },x\right\}$ for which the distribution of the statistic $S=l\left(\theta ,x\right)-l\left(\theta^{\prime },x\right)$ may be known, and to use this knowledge in turn to help to elicit a Bayes factor and therefore setting *w*. A concrete example will help.

Suppose that $\theta_{0}$ denotes the unknown mean of a population with prior *N*(0,*v*) and loss function $l\left(\theta ,x\right)=\Sigma_{i=1}^{n}.1pt{\left(\theta -x_{i}\right)}^{2}$. Consider the design points $\left\{\theta =\overset{¯}{x},\theta^{\prime }=0,x\right\}$ so that the statistic *S* is then

$$S=\sum_{i=1}^{n}x_{i}^{2}-\sum_{i=1}^{n}{\left(\overset{¯}{x}-x_{i}\right)}^{2},$$

the difference in the sum of squares to the sum of squares around the mean, with log‐Bayes‐factor

$$\log\;\left\{\frac{\pi (\theta |x)}{\pi (\theta^{\prime }|x)}/\frac{\pi (\theta )}{\pi (\theta^{\prime })}\right\}=-wS.$$

The analyst is free to contemplate any value of *n* and any $x=\{x_{1},\ldots ,x_{n}\}$ to help in the elicitation. Let *n* be chosen large and contemplate *x* such that the (1−*α*)% confidence interval for the unknown mean touches $\theta^{\prime }=0$. In this case, for large *n*, we know that $S=F_{1,\;n-1}^{-1}\left(1-\alpha \right)$, where *F* denotes the *F*‐distribution. If the analyst is prepared to say how their prior beliefs would be updated on observing *x* in knowledge of this symmetric confidence interval for $\theta_{0}$ then the *w* can be set via

w=−log(Bayes factor)/S.

We give a concrete illustration of this approach in Section 5.

### 3.5 Conjugate loss prior

If prior beliefs about *θ* can be expressed in the form

π(θ)∝exp{−λl(θ,μ)}

for given parameters (*λ*,*μ*), then the posterior has a conjugate‐type property, i.e.

π(θ|x)∝exp{−wl(θ,x)−λl(θ,μ)}.

Thus the prior has interpretation of prior observation *μ* with precision *λ*. Thus *μ* and *λ* would be standard objects for a Bayesian to specify. If the prior can then be established as the equivalent of *m* observations, then we obtain *w* via *w*/*λ*=1/*m*.

If the prior is thought not to be able to be specified in such a way, then a good approximation to any prior can be found with choices of $\left(M,\left(\mu_{j}\right),\left(\lambda_{j}\right)\right)$ such that

$$\pi \left(\theta \right)\propto \exp\;\{-\sum_{j=1}^{M}.5pt\lambda_{j}l\left(\theta ,\mu_{j}\right)\}\;.$$

If we now write

$$\pi \left(\theta |x\right)\propto \exp\;\{-wl\left(\theta ,x\right)-\Lambda \sum_{j=1}^{M}\left(\lambda_{j}/\Lambda \right)l\left(\theta ,\mu_{j}\right)\}\;,$$

where $\Lambda =\Sigma_{1⩽j⩽M}.8pt\lambda_{j}$, then we see that now *w*/Λ=1/*m*.

Thus there is an apparent new concept here in that the experimenter is required to think about how much information, in the form of the number of prior observations, is available. However, this is not completely new, since in some conjugate problems there are parameters which do have the interpretation of a prior sample size; the exponential family, for example.

## 4 General forms of information

In this section we discuss more general forms of information *x* rather than assume that it arises from some unknown $F_{0}\left(x\right)$. The argument is that provided that *l*(*θ*,*x*) has been specified then an update of a belief distribution about *θ* is available. Clearly this does not rely on any assumption about where *x* came from or indeed how it became known.

In particular, we provide a definition of conditional probability when non‐stochastic information is available. This allows for updating or refinement of prior beliefs to be applied in much more general settings than Bayesian models, which require a stochastic *x*.

### 4.1 Conditional probability distributions and non‐stochastic data

The theory of conditional probability distributions is a well‐established mathematical theory which provides a procedure to update prior probabilities taking into account new information. Such a procedure is available *only* if the information which is used to update the probability concerns stochastic events, i.e. events to which a probability is preassigned. In other words, such information needs to be already included in the probability model. In this section, we shall show how the updating approach can be used to define conditional probability distributions based on non‐stochastic information.

Information about *θ* may arrive in the form of non‐stochastic data, such as if an expert declares that

(13) $$I={}^{\prime }\theta \text{is close to}\;0^{\prime }.$$

This type of information has been discussed by various researchers and is known to be problematic for the Bayesian especially when such information arises after or during the arrival of stochastic observations $\left(x_{i}\right)$. We cite Diaconis and Zabell (1982) and in particular refer the reader to the example in section 1.1 of their paper.

We denote by *I* a piece of information for which no probability model for each *θ* is assigned; in other words *I* is not and cannot be considered stochastic in any way. So we cannot represent equation (13) by using a probability model whereby we could reasonably assume $I∼F_{0}\left(\cdot \right)$ in any meaningful sense.

Although a probability model cannot connect equation (13) and *θ*, they can be connected via a loss function without much difficulty. For example, $l\left(\theta ,I\right)=w\theta^{2}$ for some *w*>0 could be deemed appropriate. Note here that we use *I* to denote information now, replacing the stochastic *x*. The update $\hat{\nu }\left(\theta \right)$ based on *I* and *π* can then be considered as a means of defining an operational conditional probability distribution in the presence of non‐stochastic information, given by

$$\hat{\nu }\left(\theta |I\right)=\frac{\exp\{-wl(\theta ,I)\}\pi (\theta )}{\int \exp\{-wl(\theta ,I)\}\pi (d\theta )}.$$

So, the general Bayesian approach provides a general definition of conditional distributions based on non‐stochastic information, which may also be useful in the construction of priors from multiple information sources.

For literature on paradoxes related to forcing non‐stochastic events into a probability model with a determination of all the alternatives to *I* we refer the reader to Freund (1965), Gardener (1959), Bar‐Hillel and Falk (1982) and Hutchison (1999, 2008).

### 4.2 Partial information

As noted in Section 2, although the parameter of interest is *θ*, the information *I* that is collected may be more informative, i.e. there is within *I* information which does not assist with the learning about *θ*, for which it is possible to identify $I_{\theta }\subset I$ which provides *all* the information about *θ*. We are therefore interested in constructing the loss function $l(\theta ,I_{\Theta })$, leading to

(14) $$\hat{\nu }\left(d\theta \right)\propto \exp\left\{-l\left(\theta ,I_{\Theta }\right)\right\}\pi \left(d\theta \right).$$

The partial likelihood, or partial self‐information loss, that is used in proportional hazards models is one such example. Whereas Bayesian practitioners may have adopted such a procedure in the past it would be regarded as lacking motivation. However, our point is that expression (14) represents a valid update of beliefs. We illustrate this approach in Section 5.

## 5 Illustrations

In this section we discuss the application of our approach to important inferential problems. The first problem is one from survival analysis where we have a well‐motivated proxy likelihood based on partial information, and hence it is natural to use *w*=1 in this setting. The second example is from model‐free clustering where we have a general loss function so that calibration of *w* is important. A third example, which is to be found in the on‐line supplementary material, is for joint inference on a set of quantiles. In all cases we claim that the choice of loss function is well founded (and unique) and that there is no traditional Bayesian interpretation of the updates that we are implementing. Yet the updates that we employ do allow us to learn about the specified parameters of interest. All of the models that are used to generate results are available as open‐source code in R or MATLAB.

### 5.1 Colon cancer genetic survival analysis

Colon cancer is a major worldwide disease with increasing prevalence particularly within western societies. Exploring the genetic contribution to variation in survival times following incidence of the cancer may shed light into the disease aetiology and underlying disease heterogeneity. For this collaborators at the Wellcome Trust Centre for Human Genetics, University of Oxford, obtained survival times on 918 cancer patients with germline genotype data at hundreds of thousands of markers genomewide. For demonstration we consider only one chromosome previously identified as holding a potential association signal containing 15608 genotype measurements. The data table *X* then has *n*=918 rows and *p*=15 608 columns, where ${\left(X\right)}_{ij}\in \left\{0,1,2\right\}$ denotes the genotype of the *i*th individual at the *j*th marker. Alongside this we have the corresponding *n*×2 response table of survival times *Y* with a column of event times, $y_{i1}\in R^{+}$ and a column of indicator variables $y_{i2}\in \left\{0,1\right\}$, denoting whether the event is observed or right censored at $y_{i1}$.

To explore association between genetic variation and time to event we employ a loss function derived under proportional hazards, treating the loss to the baseline hazard as a nuisance parameter. This is based on the Cox proportional hazards model, which has been one of the most widely used methods in survival analysis since its introduction in Cox (1972). In this log‐linear model the hazard rate at time *t* for an individual with covariate $x=\{x_{1},\ldots ,x_{p}\}$ is defined as

$$h\left(t|x\right)=h_{0}\left(t\right).1pt\exp\;\;(\sum_{j=1}^{p}.2ptx_{j}\beta_{j})$$

where $h_{0}\left(t\right)$ is a baseline hazard function. In the seminal work of Cox (1972), $h_{0}\left(t\right)$ is treated as a nuisance parameter (or process) that does not enter the partial likelihood for estimating the parameters of interest ***β***.

In contrast, a Bayesian approach to the Cox model necessarily involves the baseline hazard function. There is a limiting argument for the use of the partial likelihood but this is rarely, if at all, used. Most common is the finite partitioning of the time axis and using a piecewise constant baseline hazard function. Though typically regarded as a nuisance parameter, the Bayesian must specify a full probability model for it. See Ibrahim *et al*. (2001), chapter 3, for details, where they noted that the proportional hazards model is obtained under a limiting improper prior on the baseline, but it is not known what effect this has on marginal quantities of interest such as marginal model choice probabilities.

Using a general Bayes construction we can consider only the order of events as partial information relevant to the regression coefficients ***β***, via the cumulative loss function,

(15) $$l\left(\beta ,x\right)=\sum_{i=1}^{n}\log\;\left\{\frac{\exp\;\;(\sum_{j=1}^{p}x_{ij}\beta_{j})}{\sum_{l\in R_{i}}\exp\;\;(\sum_{j=1}^{p}x_{lj}\beta_{j})}\right\}\;\;,$$

where $R_{i}$ denotes the risk set, i.e. those individuals alive or not censored at time $y_{i1}$, and in this way obtain a conditional update. We assume that $\beta_{j}∼N\left(0,v_{j}\right)$ and set $v_{j}=0.5$ for our study, reflecting beliefs that associated coefficients will be modest, and we note that one advantage of our approach is that subjective prior information can be integrated into the analysis.

Initially we consider each marker in turn for evidence of effects, i.e. $\beta_{j}\neq 0$, within a univariate regression and we can calculate the general Bayes factor of association at the *j*th marker, assuming equal prior probability in there being an effect or not, as,

$${\text{gBF}}^{\left(j\right)}=\frac{\int_{\;\;\;\beta_{j}}\exp\left\{-l\left(\beta_{j}|x_{j}\right)\right\}.5pt\pi \left(\beta_{j}\right)d\beta_{j}}{\exp\{-l\left(\beta_{j}=0|x_{j}\right)\}}$$

which involves a one‐dimensional integral that we calculate via importance sampling.

**Figure 1:**
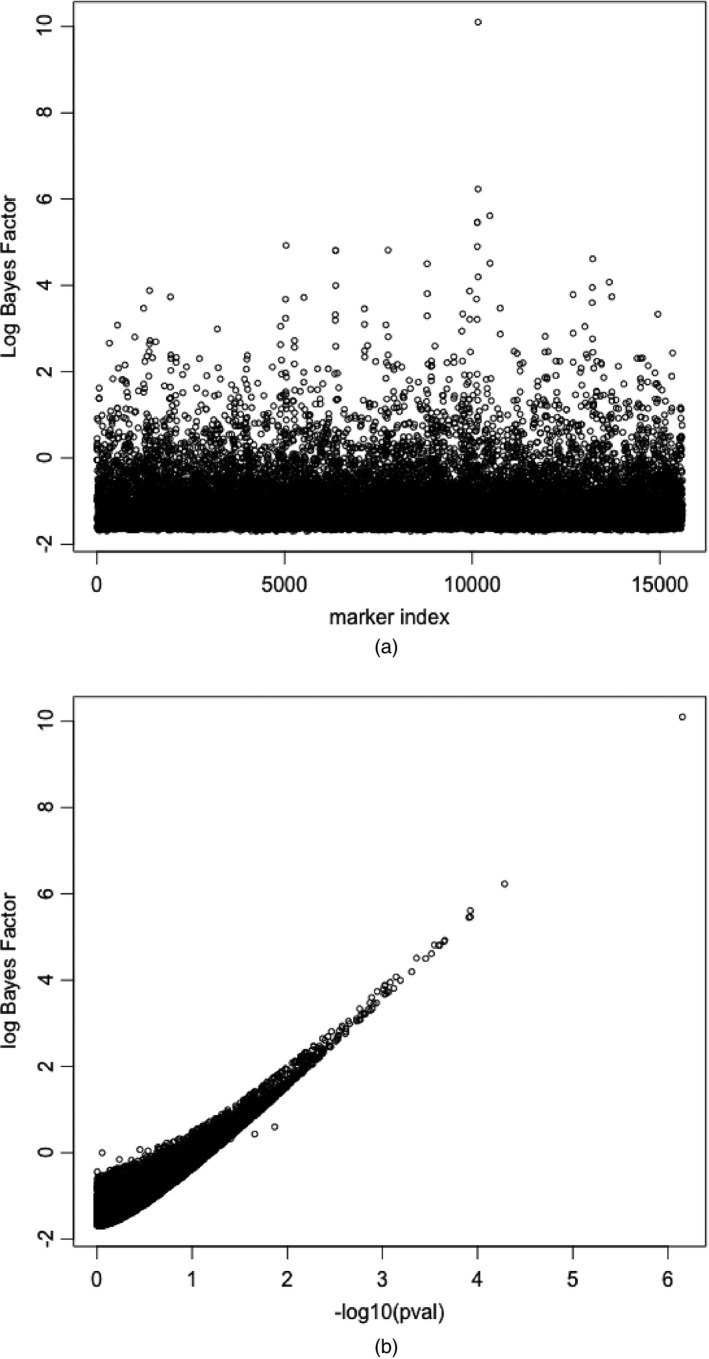
(a) log‐Bayes‐factor *versus* marker index and (b) log‐Bayes‐factor *versus* ${\text{log}}_{10}$(*p*‐value) of association

**Figure 2:**
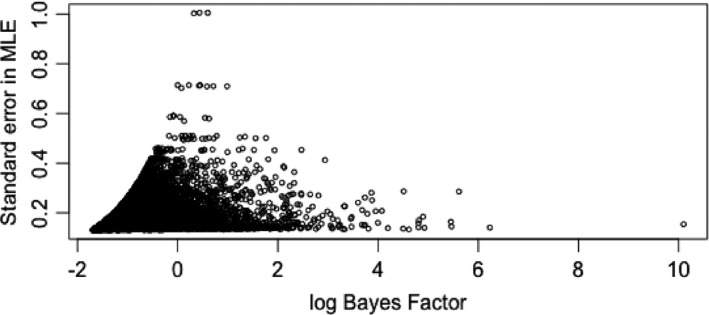
Standard error in maximum likelihood estimate *versus* log‐Bayes‐factor

We calculated the general Bayes factors for each marker and in Fig. 1(a) we plot the log‐general‐Bayes factors over the chromosome. Although there is considerable variation we observe strong evidence of association around marker 10000. It is interesting to compare the evidence of association that is provided by the Bayes factor Fig. 1(a) with that obtained by using a conventional Cox proportional hazards partial‐likelihood‐based test. In Fig. 1(b) we plot the log‐general‐Bayes factors against $-\log_{10}$(*p*‐values) obtained from a conventional likelihood ratio test at each marker. We can see general agreement especially at the markers with strongest association as we would expect for a large sample size. Interestingly there appears to be greater dispersion at markers of weaker association as highlighted in Fig. 2 where we plot the standard error against log‐general‐Bayes factors. Markers with high standard error relate to genotypes of rarer alleles and the attenuation reflects a greater degree of uncertainty for association at these markers that contain less information.

Returning to the ‘hit region’ showing strongest association around marker 10000, owing to high collinearity between markers it is not clear whether the signal of association arises from a single effect correlated with others, or from multiple independent association signals. To investigate this we developed multiple‐marker methods.

We consider a model using potentially all 800 markers in the region and phrase the problem as a variable selection task under a partial likelihood (loss), in which the user suspects that some of the *p*=800 recorded covariates (15) may not be relevant to variation in survival times.

In the non‐Bayesian paradigm, variable selection can proceed by defining a cost function, such as the Akaike information criterion AIC or Bayesian information criterion BIC, that adjusts the fit to the data by the number of covariates in the model. Inference proceeds by using an optimization algorithm, such as forward or stepwise selection, to find a model that minimizes the cost. More recently, penalized likelihood methods have proved popular (Tibshirani, 1997; Fan and Li, 2002).

Despite the enormous influence of Cox proportional hazards models and the importance of variable selection, the Bayesian literature in this area is limited. This is because of the lack of a theoretical foundation to treat $h_{0}\left(t\right)$ as a nuisance parameter, leading to either approximate methods or the full specification of a joint probability model (Faraggi and Simon, 1998; Volinsky *et al*., 1997). Volinsky *et al*. (1997) took BIC as an approximation to the marginal likelihood and they used a branch‐and‐bound algorithm to find a set of models with differing sets of covariates with high BIC‐scores. The difficulty here is that, although the methods are important and well motivated, they are ultimately *ad hoc*. Moreover, prior information on *π*(***β***) does not enter the calculation of BIC, meaning that an important aspect of the Bayesian approach is lost.

In contrast, Ibrahim *et al*. (1999) considered variable selection within a full joint model using a prior specification of a gamma process for the baseline hazard (see also Ibrahim *et al*. (2001)). This provides a formal Bayesian solution but inference is then conditional on, and sensitive to, the specification of the prior on $h_{0}\left(t\right)$, which is something that the partial likelihood model explicitly avoids.

Here we use the partial information that is relevant to the regression coefficients ***β*** via the cumulative loss function (15). We assume proper priors *π*(***β***) on the regression coefficient,

$$\pi \left(\beta_{j}\right)=\left\{\begin{array}{cc} 0 & \text{if}\;\delta_{j}=0,\\ N(0,v_{j}) & \text{otherwise}, \end{array}\right.$$

where $\delta_{j}\in \left\{0,1\right\}$ is an indicator variable on covariate relevance with $\pi \left(\delta_{j}\right)=\text{Bin}\left(a_{j}\right)$ and we now treat $\left\{\delta_{1},\ldots ,\delta_{800}\right\}$ as a vector in a joint model. In this way the posterior *π*(***δ***|**x**) quantifies beliefs about which variables are important to the regression. We use Markov chain Monte Carlo (MCMC) sampling to draw samples approximately from *π*(***β***,***δ***|**x**) from which the marginal distribution on ***δ*** can be examined. In particular we make use of an efficient joint updating proposal, $q(\delta^{\prime },\beta^{\prime }|\delta )$, within the MCMC algorithm as $q\left(\delta^{\prime },\beta^{\prime }|\delta \right)=q\left(\delta^{\prime }|\delta \right)q\left(\beta^{\prime }|\delta^{\prime }\right)$ where $q(\delta^{\prime }|\delta )$ proposes a local move to add, remove or swap one variable per MCMC iteration in or out of the current model indexed by ***δ***, and $q(\beta^{\prime }|\delta^{\prime })$ is a joint independence Metropolis update proposal, $q\left(\beta^{\prime }|\delta^{\prime }\right)=N\left({\tilde{\beta }}_{\delta^{\prime }},{\tilde{V}}_{\delta^{\prime }}\right)$ where $\left\{{\tilde{\beta }}_{\delta^{\prime }},{\tilde{V}}_{\delta^{\prime }}\right\}$ are the maximum *a posteriori* and approximate information matrix obtained from the combination of log‐partial‐loss and normal prior. The joint proposal is then accepted with probability

$$\alpha =\min\;\;\left[1,\frac{\exp\left\{-l\left(\beta^{\prime }|x\right)\right\}\pi \left(\beta^{\prime }|\delta^{\prime }\right)\pi \left(\delta^{\prime }\right)q\left(\beta ,\delta |\delta^{\prime }\right)}{\exp\left\{-l\left(\beta |x\right)\right\}\pi \left(\beta |\delta \right)\pi \left(\delta \right)q\left(\beta^{\prime },\delta^{\prime }|\delta \right)}\right]\;\;.$$

**Figure 3:**
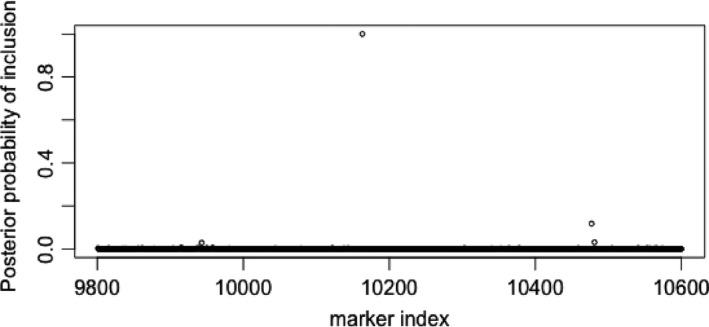
Posterior marginal inclusion probability from the multiple‐marker model using 800 markers around the peak of association

We ran our MCMC algorithm for 100000 iterations with prior parameter settings, $\left\{v_{j}=0.5,a_{j}=1/800\right\}$, for all *j*=1,…,*p*, equivalent to a prior assumption of a single associated marker. In Fig. 3 we show the marginal inclusion probability, after discarding 10000 samples as a burn‐in. The algorithm showed an overall acceptance rate of 8% for proposed moves. The model suggests overwhelming evidence for a single marker in the region of index 10200 but also weaker evidence of independent signal in a couple of other regions.

### 5.2 Bayesian model‐free clustering

Cluster analysis is one of the most widely used and important areas of applied statistics (Hastie *et al*., 2009). In cluster analysis, a primary objective is to identify self‐similar groups within data such that observations within a group are deemed more closely related to one another than observations between groups, *K*‐means clustering being arguably the most popular clustering method in use today.

The clustering problem is interesting from a formal Bayesian perspective as it raises several challenges. The object of interest is the cluster partition mapping *S*, which allocates observations to clusters. However, the partition *S* as it stands is not a generative model (a sampling distribution for observables). To implement clustering the Bayesian analyst is forced to define a sampling distribution for observations within a cluster, $f(x|C_{j})$, where $C_{j}$ denotes parameters that are associated with the *j*th cluster, with an associated prior probability of cluster membership $p_{j}$. This leads to the well‐known marginal mixture representation

$$f\left(x|C\right)=\sum_{j=1}^{K}.5ptp_{j}.8ptf_{j}\left(x|C_{j}\right),$$

the canonical example being with Gaussian mixture components, $f\left(x|C_{j}\right)=N\left(\mu_{j},\Sigma_{j}\right)$, which necessitates a further layer of hierarchical priors $\pi (\mu_{j},\Sigma_{j})$. Cluster membership can be sensitive to the choice of sampling distribution and hierarchical prior, both of which are nuisance to the task, and computation is complicated by the well‐known label switching problem (Jasra *et al*., 2005).

Non‐Bayesian model‐free segmentation methods have a distinct advantage in allowing the analyst to concentrate on the object of interest, namely the clustering *S*, typically defined through the specification of a pairwise dissimilarity score between observations $d(x_{i},x_{j})$. An optimization algorithm is then used to find the optimal partition $\hat{S}$ which minimizes the score over pairs within and/or between clusters. However, quantifying uncertainty in $\hat{S}$, even assuming that the global minima can be found, is far from trivial as we typically have only a single realization of dependent multivariate data *x*, although see Seldin and Tishby (2001), who used PAC Bayesian ideas to consider uncertainty in a predictive regression model when clustering the covariates.

**Figure 4:**
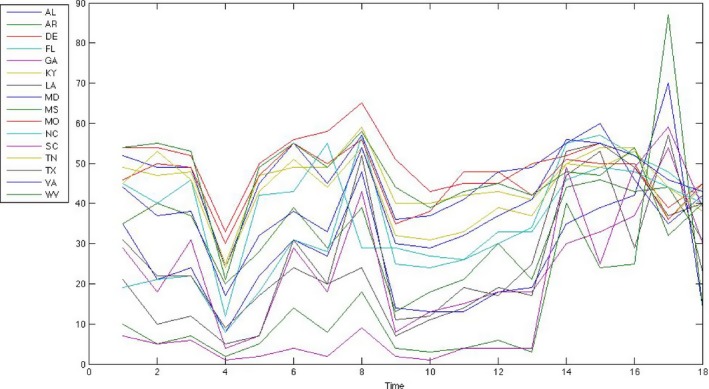
Voting of southern states, illustrating the percentage of the Republican vote for Presidential elections every 4 years beginning in 1900: AL, Alabama; AR, Arkansas; DE, Delaware; FL, Florida; GA, Georgia; KY, Kentucky; LA, Louisiana; MD, Maryland; MS, Mississippi; MO, Missouri; NC, North Carolina; SC, South Carolina; TN, Tennessee; TX, Texas; VA, Virginia; WV, West Virginia

We define a prior distribution directly on the partition, *π*(*S*), and a loss function $l(S,x_{1},\ldots ,x_{n})$ and we use general Bayesian updating. To illustrate this we consider uncertainty analysis of a classic data set considered in Hartigan (1972), illustrated in Fig. 4, in his highly influential paper that introduced biclustering. Biclustering refers to the simultaneous clustering of observations and covariates (rows and columns) of a data matrix and has proved extremely useful in modern application areas, particularly in genomics (Cheng and Church, 2000; Tanay *et al*., 2002; Heard *et al*., 2005).

Hartigan (1972) considered the percentage Republican Presidential vote of 16 southern states in the USA over 18 elections covering the years 1900–1968. Hartigan treated the time series as independent covariates in his co‐clustering approach. Here, for simple illustration, we maintain the time series ordering, so that the co‐clustering is akin to clustering multiple‐change‐point time series with common but unknown change points. We assume that the cluster memberships are constant over time, but the time series change at specific break points. Our loss function is defined as in Hartigan (1972) using a sum‐of‐squares decomposition,

$$l\left(S,x_{1},\ldots ,x_{n}\right)=w\sum_{C_{k}\in S}.8pt\sum_{ij\in C_{k}}.8pt{\left(x_{ij}-{\overset{¯}{x}}_{C_{k}}\right)}^{2},$$

where *i* denotes state and *j* denotes time, $C_{k}$ denotes the *k*th grouping of states over a particular time period and ${\overset{¯}{x}}_{C_{k}}$ denotes the mean over all $\left(i,j\right)\in C_{k}$. The posterior distribution is therefore

P(S|x)∝π(S).5ptexp{−l(S,x)}.

The setting of the loss parameter *w* is a crucial part of the model specification. Following the procedures that were discussed in Section 3, it is difficult to consider a conjugate specification or a unit information prior on the discrete structures. We instead propose to use a frequentist calibration approach in the following manner. Recall that under a flat prior on *S* we can set *w* via a subjective assessment of the posterior ratio at a reference point,

$$\frac{P(S|x)}{P(S^{\prime }|x)}=\exp\left[-w\left\{l\left(S,x\right)-l\left(S^{\prime },x\right)\right\}\right],$$

and where we can solve for *w* if all the other elements are given. In elicitation of *w* we propose to make use of classical results from analysis of variance. We take as our first reference point the null partition using a single global cluster, so that the loss $l\left(S,x\right)=\Sigma_{i}.8pt\Sigma_{j}.5pt{\left(x_{ij}-\overset{¯}{x}\right)}^{2}$ is simply the sum of squares around the mean. Then consider a randomized data partition $\left\{x,S^{\prime }\right\}$ that allocates the data uniformly at random to *k* clusters. Under this scheme we expect that

$$\frac{\{l\left(S,x\right)-l\left(S^{\prime },x\right)\}/k-1}{l(S,x)/(n-k)}∼F_{k-1,\;n-k}$$

where *F* denotes the *F*‐distribution. We can then use the *F*‐distribution to help in the calibration. For example, if we consider a point in the tails of *F*, such that $f_{\alpha }^{*}=F_{k-1,\;n-k}^{-1}\left(\alpha \right)$ with *α* ∈ (0,1), and specify

$$l\left(S^{\prime },x\right)=\frac{l(S,x)}{1+f^{*}\left(k-1\right)/\left(n-k\right)}$$

then $l(S^{\prime },x)$ represents the value of loss such that a randomized allocation has probability 1−*α* of producing a smaller loss. Equivalently, with probability *α* a random allocation would lead to a reduction in loss as high as $1+f_{\alpha }^{*}\left(k-1\right)/\left(n-k\right)$ relative to the single cluster. When *α* is large we can be confident that a partition achieving a loss of $l(S^{\prime },x)$ represents a significant clustering. The analyst can then calibrate *w* in the following way.

- Define a reference value for $R=P\left(S|x\right)/P\left(S^{\prime }|x\right)$ under a uniform prior, setting *R* small, say *R*=0.001, relative to the global cluster *S*.
- Define a tail area value *α* such that, should a partition $S^{\prime }$ achieve a relative reduction in loss of $1+f_{\alpha }^{*}\left(k-1\right)/\left(n-k\right)$, then you would assign relative posterior beliefs of *R*.
- Set $w=-\log\left(R\right)l\left(S,x\right)f_{\alpha }^{*}\left(k-1\right)/\left(n-k\right)$.

For the election data we found that *w* is quite stable to the calibration choice of {*α*,*R*}; for example with *k*=3 we find *w*=0.0036 for {*α*=0.99,*R*=0.01} and *w*=0.0012 for {*α*=0.999,*R*=0.01}. We choose *w*=0.0012 and ran an MCMC algorithm for 100000 iterations using a burn‐in of 50000. The iteration numbers were chosen after experimentation to deliver stable results over multiple runs. The MCMC algorithm was rerun for differing numbers of partitions of states and differing number of time series change points. Table 1 presents the results of the average loss achieved over each run alongside the estimate of the posterior probability for each configuration shown in parentheses by using a Poisson(3) prior on the number of groups and a Poisson(2) prior on the number of *time groupings*, which is the number of change points plus 1. Note that the first column in Table 1 equates to standard clustering of the states with zero change points in time, whereas the first row represents a multivariate change point model. Table 1 suggests strong evidence for clustering in both time and across states. The maximum posterior probability favours the model with three groups of states and three time groupings.

**Table 1:** Average loss of partitions across MCMC samples (and log‐posterior probabilities in parentheses)†

*Number of state*	*Average loss* $\times 10^{4}$ *for the following numbers of*		
*clusters* $k_{s}$	*change points in time* $k_{t}$ *(groups* $=k_{t}+1)$		
	$k_{t}=0$	$k_{t}=1$	$k_{t}=2$
1	7.98 (−14.49)	6.82 (−14.34)	6.72 (−14.73)
2	5.36 (−13.69)	5.13 (−13.65)	3.19 (−13.58)
3	5.09 (−13.64)	3.92 (−13.38)	2.36 (−13.28)
4	4.99 (−13.91)	3.32 (−13.50)	2.02 (−13.41)

†The average loss is $T^{-1}\Sigma_{i=1}^{T}l\left(S_{i},x\right)$ with $S_{i}∼\pi \left(S|x,k_{s},k_{t}\right)$, where $k_{s}$ denotes the number of clusters of states and $k_{t}$ denotes the number of time series change points. Log‐posterior‐probabilities are shown in parentheses using a Poisson(3) and Poisson(2) prior on the number of groups and number of time clusters $k_{t}+1$. The maximum posterior clustering is shown in italics.

**Figure 5:**
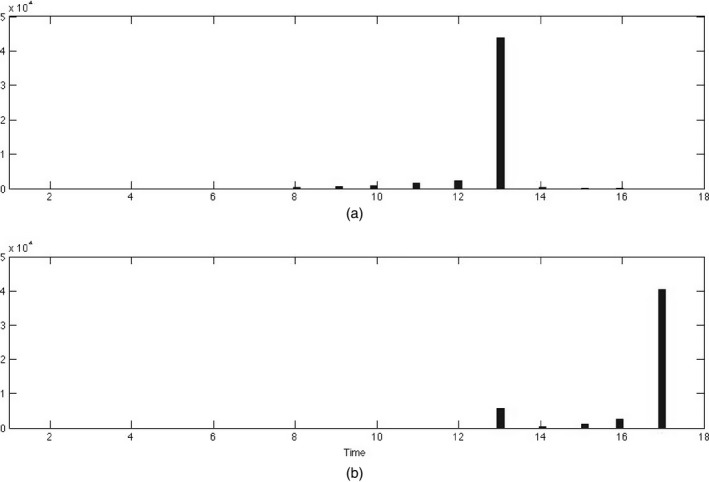
Time change point locations for the two‐change‐point, $k_{t}=2$, model and $k_{s}=3$ groups: (a) change point 1; (b) change point 2

**Figure 6:**
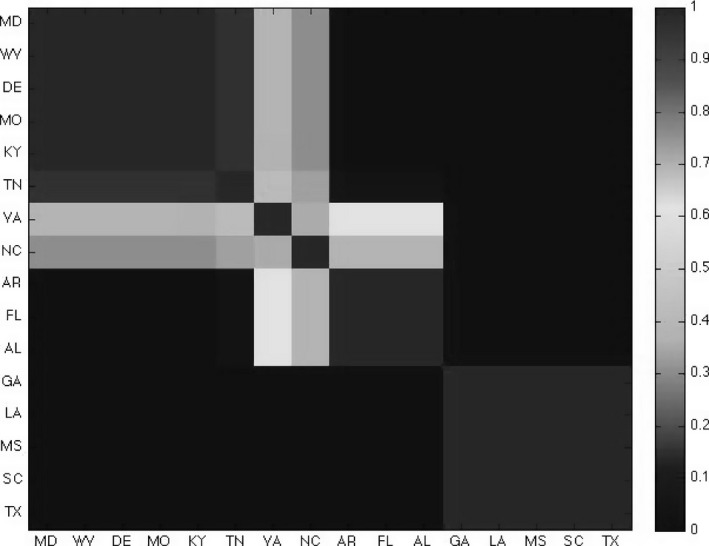
Pairwise co‐clustering probabilities across three groups and two time change points: AL, Alabama; AR, Arkansas; DE, Delaware; FL, Florida; GA, Georgia; KY, Kentucky; LA, Louisiana; MD, Maryland; MS, Mississippi; MO, Missouri; NC, North Carolina; SC, South Carolina; TN, Tennessee; TX, Texas; VA, Virginia; WV, West Virginia

We investigated uncertainty in the partitions and in the cluster allocation of the maximal posterior model. To illustrate this we plot in Fig. 5 the distribution of the location of time series change points for the $\left\{k_{s}=3,k_{t}=2\right\}$ model. We can see strong evidence that the change points occur late in the series, which is visually supported by the data in Fig. 4. The pairwise co‐clustering probabilities of this model are shown in Fig. 6, where each element represents the pairwise probability events $\Sigma_{S}.5ptP\left(S|x\right)1\left[\text{CI}\left(x_{i}\right)=\text{CI}\left(x_{j}\right)|S\right]$, where $\text{CI}(x_{i})$ is the cluster index for the *i*th state. The cluster blocks show strong concordance with the single co‐cluster that was reported by Hartigan; see Fig. 6(a) in Hartigan (1972). However, our method highlights considerable uncertainty in the pairing of Virginia and North Carolina, which is something that we can quantify by using our general Bayesian approach.

## 6 Discussion

We have provided a basis for general Bayesian learning and the updating of information by using belief probability distributions. Loss functions constructed on spaces of probability measures allow for coherent updating. Specifically, information is connected to the parameter of interest via a loss function and this is the fundamental concept, replacing the restrictive connection based on probability models. We can recover precisely the traditional updating rules such as the Bayes rule when we select the self‐information loss function, when it is appropriate to do so.

The assumptions that we make are minimal: that information can be connected to unknown parameters via loss functions and that individuals then act rationally by minimizing their expected loss. If information is assumed to come from some probability model then we can accommodate this within our framework by appealing to the self‐information loss function equivalent to the negative log‐likelihood and so we can argue that loss functions are sufficient for learning mechanisms that are currently in use.

More generally, we can use loss functions that are currently employed in a classical context for robust estimation, e.g. generalized estimating equations. We can also deal with partial information where it is only a part of some observed information that is useful or relevant for learning about the decision‐making process based on a particular relevant parameter of interest.

We have developed a rigorous approach to updating beliefs where we are required only to think about which is the best parameter from a chosen model needed to make a decision rather than have to think about a non‐existent true model parameter which coincides with the true data‐generating mechanism.

We believe that it is fundamental to identify parameters of interest through loss functions. The alternative route through a probability model is, we argue, highly restrictive and leads to narrow types of Bayesian updating. The necessary supporting theory for us is minimal (the construction and minimization of loss functions), whereas for the use of probability models it is more intricate and restrictive.

## Supporting information

‘Supplementary material’.

## Appendix A Proof of theorem 1

In the two‐point case, say *Θ*={0,1}, the prior *π* is determined by a real number *z* in the unit interval, and the loss *l*(*θ*,*x*) takes only two values, say $l_{0}$ and $l_{1}$. By condition 5, we can replace the pair $\left(l_{1},l_{0}\right)$ with $\left(l_{1}-l_{0},0\right)$. Therefore, the posterior is a function of $l=l_{1}-l_{0}$ and *z*, say $\overset{¯}{\psi }(l,z)$. To proceed, it is convenient to think in terms of odds rather than probabilities. So, if *z* is the prior for 0, we can consider *t*=*z*/(1−*z*) and *z*=*t*/(1+*t*). We can do the same with the posterior. The posterior odds are a function of the loss difference *l* and the prior odds *t*, which we denote by *ϕ*(*l*,*t*).

Dealing with the odds, equation (3) becomes

(16) ϕ(l+h,t)=ϕ{l,ϕ(h,t)},

where *h* is a replication of *l* with a possibly different *x*. Moreover, with a constant loss, the posterior is equal to the prior (condition 4), i.e.

(17) ϕ(0,t)=t,

for every *t*>0.

At this stage, we consider a prior with more than two mass points, say three and given by {1,2,3}; the prior is given by $\left\{z_{1},z_{2},1-z_{1}-z_{2}\right\}$, with loss functions $\left\{l_{1}\left(x\right),l_{2}\left(x\right),l_{3}\left(x\right)\right\}$. The loss can be 0 at one point without loss of generality (condition 5), so it takes values $\left(l_{1},l_{2},0\right)$. Let us consider the updating rule *ϕ* for priors with just two mass points. We can use this to update the conditional probability of 1 given {1,3}, i.e. $z_{1}/\left(z_{1}+z_{3}\right)$.

For this, we update the prior with masses $z_{1}/\left(z_{1}+z_{3}\right)$ and $z_{3}/\left(z_{1}+z_{3}\right)$ considering just the loss values $\left(l_{1},0\right)$, i.e. disregarding the point 2 with its loss $l_{2}$. In other words, we aim at the right‐hand side of equation (9).

Denote by $t_{1,\;3}$ the odds corresponding to the conditional probability of 1 given {1,3}, i.e. $t_{1,\;3}=z_{1}/z_{3}$. We update $t_{1,\;3}$ on the basis of the loss values $l_{1}$ and 0, i.e. with $\phi (l_{1},t_{1,\;3})$. Similarly, we can define $t_{1,\;2}=z_{1}/z_{2}$ and $t_{2,\;3}=z_{2}/z_{3}$. We update $t_{1,\;2}$ on the basis of $l_{1}$ and $l_{2}$, i.e. with $\phi (l_{1}-l_{2},t_{1,\;2})$ and $t_{2,\;3}$ with $\phi (l_{2},t_{2,\;3})$. Clearly, $t_{1,\;3}=t_{1,\;2}t_{2,\;3}$, and this factorization of conditional odds must hold also after updating, i.e.

(18) $$\phi \left(l_{1},t_{1,\;3}\right)=\phi \left(l_{1}-l_{2},t_{1,\;2}\right)\phi \left(l_{2},t_{2,\;3}\right),$$

where $t_{1,\;3}=t_{1,\;2}t_{2,\;3}$. Formally, this identity is a consequence of condition 2, i.e. updating the conditional probability is the same as conditioning the updated probability. Since equation (17) must hold for every $t_{2,\;3},t_{1,\;2}>0$ and for every $l_{1},l_{2}\in R$ we have that

$$\phi \left(l_{1},ts\right)=\phi \left(l_{1}-l_{2},t\right)\phi \left(l_{2},s\right),$$

for every *t*,*s*>0 and $l_{1},l_{2}\in R$. If in particular $l_{2}=0$, and say $l_{1}=l$, recalling equation (16), we have that

ϕ(l,ts)=ϕ(l,t)s,

for every *t*,*s*>0 and every real *l*. Letting *t*=1, we find that

(19) ϕ(l,s)=ϕ(l,1)s,

for every *s*>0 and every l∈R. A combination of equations (16) and (19) yields *ϕ*(*l*+*h*,1)=*ϕ*{*l*,*ϕ*(*h*,1)}=*ϕ*(*l*,1) *ϕ*(*h*,1), for every h,l∈R, which in turn, it is known, implies that *ϕ*(*l*,1)= exp (−*wl*) for some w∈R, provided that *ϕ*(*l*,1) is a monotone function of *l* owing to condition 3. Hence,

(20) ϕ(l,t)=exp(−wl)t,

for every l∈R and every *t*>0. In this way, we are basically done with the two‐points case.

Let us now consider the three‐point case: we want to update the prior with mass points at {1,2,3} given by $\left\{z_{1},z_{2},1-\left(z_{1}+z_{2}\right)\right\}$, where $z_{1}$ and $z_{2}$ are non‐negative and their sum is less than or equal to 1, and it is convenient to set $z_{3}:=1-\left(z_{1}+z_{2}\right)$. In terms of odds, the prior is given by the pair $\left(t_{1},t_{2}\right)$ (being $t_{i}=z_{i}/\left(1-z_{i}\right)$, or equivalently $z_{i}=t_{i}/\left(1+t_{i}\right)=1-1/\left(1+t_{i}\right)$, *i*=1,2) where $t_{1},t_{2}\;⩾\;0$ and $t_{1}/\left(1+t_{1}\right)+t_{2}/\left(1+t_{2}\right)⩽1$. Here, it is convenient to set $t_{3}:={\left[{\left\{1-t_{1}/\left(1+t_{1}\right)+t_{2}/\left(1+t_{2}\right)\right\}}^{-1}-1\right]}^{-1}$. In this setting, we consider the loss values $\left(l_{1},l_{2},0\right)$. Moreover, we consider an $R^{2}$‐valued function $\phi \left(l,t\right)=(\phi_{1}\left(l,t\right),\phi_{2}\left(l,t\right)),$ where $l=(l_{1},l_{2})$ and $t=(t_{1},t_{2})$. Now the question is how we could recover ***ϕ*** from *ϕ*, where the latter gives the updating rule for the two‐points case. Recall the notation that we have used for the conditional odds, i.e. $t_{i,\;j}=z_{i}/z_{j}$ are the odds corresponding to the conditional probability of *i* given {*i*,*j*}, for distinct *i*,*j*=1,2,3. We can see that $t_{1}=z_{1}/\left(z_{2}+z_{3}\right)={\left(t_{2,\;1}+t_{3,\;1}\right)}^{-1}$. By condition 2, this identity will have to be satisfied also by the updated odds. Since we update $t_{2,\;1}$ with $\phi (l_{2}-l_{1},t_{2,\;1})$ and $t_{3,\;1}$ with $\phi (-l_{1},t_{3,\;1})$, we must have

$$\phi_{1}\left(l_{1},l_{2};t_{1},t_{2}\right)={\left\{\phi \left(l_{2}-l_{1},t_{2,\;1}\right)+\phi \left(-l_{1},t_{3,\;1}\right)\right\}}^{-1},$$

which by equation (19) becomes

(21) $$\phi_{1}\left(l_{1},l_{2};t_{1},t_{2}\right)=\exp\left(-wl_{1}\right){\left\{\exp\left(-wl_{2}\right)t_{2,\;1}+t_{3,\;1}\right\}}^{-1}.$$

As $t_{2,\;1}=z_{2}/z_{1}$ and $t_{3,\;1}=z_{3}/z_{1}$, equation (21) becomes

$$\phi_{1}\left(l_{1},l_{2};t_{1},t_{2}\right)=\frac{\exp\left(-wl_{1}\right)z_{1}}{\exp\left(-wl_{2}\right)z_{2}+z_{3}},$$

and the updated probability of 1 will be

$$1-{\left\{1+\phi_{1}\left(l_{1},l_{2};t_{1},t_{2}\right)\right\}}^{-1}=\frac{\exp\left(-wl_{1}\right)z_{1}}{\exp\left(-wl_{1}\right)z_{1}+\exp\left(-wl_{2}\right)z_{2}+z_{3}}.$$

This is what we must obtain updating $z_{1}$ on the basis of the loss values $l_{1},l_{2}\;\text{and}\;l_{3}$ with $l_{3}=0$. Similarly, we can see that updating $z_{2}$ we must obtain

$$\frac{\exp\left(-wl_{2}\right)z_{2}}{\exp\left(-wl_{1}\right)z_{1}+\exp\left(-wl_{2}\right)z_{2}+z_{3}}.$$

In this way we have shown how to extend our coherent updating rule from the two‐points case to the three‐points case.
