# Supplementary material for: A general framework for updating belief distributions
Source: J R Stat Soc Series B Stat Methodol. 2016 Feb 23;78(5):1103–30. doi: 10.1111/rssb.12158 (PMC5082587; doi:10.1111/rssb.12158)
Supplement: Supplementary file 1 — ‘Supplementary material’. [file RSSB-78-1103-s001.pdf]

Supplementary Material

The Kullback-Leibler divergence. Here we discuss the choice of the Kullback–Leibler divergence as being appropriate for quantifying the loss-to-prior $h_2(\nu, \pi)$. With n independent pieces of information $x = (x_1, \ldots, x_n)$ we take the cumulative loss as

$$L(\nu; \pi, x) = \sum_{i=1}^{n} h_1(\nu, x_i) + h_2(\nu, \pi), \tag{1}$$

where h_1 will be taken as the expected loss:

$$h_1(\nu, x_i) = \int_\Theta l(\theta, x_i)\ \nu(\mathrm{d}\theta).$$

This is the only coherent choice based on the problem posed by minimizing

$$\int l(\theta, x)\, \mathrm{d}F_0(x).$$

Adhering to the "likelihood principle" (see Bernardo and Smith 1994), for any $0 < m < n$, all the information contained in $(x_1, \ldots, x_m)$ is to be found in $\widehat{\nu}_m$, where $\widehat{\nu}_m$ minimizes

$$L(\nu; \pi, x_1, \ldots, x_m) = \sum_{i=1}^{m} h_1(\nu, x_i) + h_2(\nu, \pi).$$

and hence it follows that,

$$L(\nu; \pi, x) = \sum_{i=m+1}^{n} h_1(\nu, x_i) + h_2(\nu, \widehat{\nu}_m),$$

where $\widehat{\nu}_m$ now serves as the prior for future information $(x_{m+1}, \ldots, x_n)$. For coherence, the solution from L for all cases of m must be the same. To derive the form of h_2 to guarantee this we start with the family of g–divergences, that is

$$h_2(\nu, \pi) = d_g(\nu, \pi) = \int g(\mathrm{d}\pi/\mathrm{d}\nu)\, \mathrm{d}\nu \tag{2}$$

where g is a convex function from $(0, \infty)$ to the real line and $g(1) = 0$. See Ali and Silvey (1966). This provides a large class of functions; and some special cases include $g(s) = 1 - \sqrt{s}$, the Hellinger divergence, which is equivalent to the L_1 metric; $g(s) = s^{-1} - 1$ yields the chi-squared divergence. For the coherence to be in force, it is necessary that the discrepancy h_2 is the Kullback-Leibler divergence; i.e. $g(s) = -\log s$. The following theorem can be stated:

Theorem. *Let the loss* $L(\nu; \pi, [x_1, x_2])$ *be defined by* (1) *and* (2)*. Moreover, let* $\widehat{\nu}_{(\pi,x_1,x_2)}$ *be the probability measure that minimizes the loss*

$$L(\nu; \pi, [x_1, x_2])$$

among the probability measures on Θ *that are absolutely continuous with respect to* π*. Similarly, let* $\widehat{\nu}_{(\pi,x_1)}$ *and* $\widehat{\nu}_{(\widehat{\nu}_{(\pi,x_1)},x_2)}$ *be the probability measures minimizing the loss* $L(\nu; \pi, x_1)$ *and* $L(\nu; \widehat{\nu}_{(\pi,x_1)}, x_2)$*, respectively. Assume that*

$$\widehat{\nu}_{(\widehat{\nu}_{(\pi,x_1)},x_2)} = \widehat{\nu}_{(\pi,[x_1,x_2])} \tag{3}$$

for every probability measure π *on* Θ *and for every choice of the loss functions* $h_1(\nu, x_1)$ *and* $h_2(\nu, x_2)$ *such that* $\widehat{\nu}_{(\pi,[x_1,x_2])}$, $\widehat{\nu}_{(\pi,x_1)}$, $\widehat{\nu}_{(\widehat{\nu}_{(x_1)},x_2)}$, *are all properly defined. Then* h_2 *is the Kullback–Leibler divergence.*

This theorem has been proven by Bissiri and Walker (2012b). A more concise proof is given, assuming the differentiability of g, here. In virtue of this Theorem, for coherence it is required to take

$$h_2(\nu, \pi) = d_{KL}(\nu, \pi) = \int \nu \, \log(\nu/\pi),$$

the Kullback–Leibler divergence. So, in the case of $m = 0$, we have

$$L(\nu; \pi, x) = \sum_{i=1}^{n} h_1(\nu, x_i) + d_{KL}(\nu, \pi),$$

where π is the initial choice of probability measure representing beliefs about θ in the absence of x.

The solution to this minimization problem is easy to find and is given by

$$\nu(\mathrm{d}\theta) = \frac{\exp\{-\sum_{i=1}^{n} l_i(\theta, x_i)\} \; \pi(\mathrm{d}\theta)}{\int \exp\{-\sum_{i=1}^{n} l_i(\theta, x_i)\} \; \pi(\mathrm{d}\theta)},$$

and this is the solution since one can see that

$$\begin{aligned} \textstyle\int l(\theta, x) \, \nu(\mathrm{d}\theta) \quad & + \textstyle\int \nu(\mathrm{d}\theta) \; \log\{\nu(\theta)/\pi(\theta)\} \\ & = \textstyle\int \nu(\mathrm{d}\theta) \; \log\{\nu(\theta)/[\exp(-l(\theta, x)) \, \pi(\theta)]\}. \end{aligned}$$

The solution is clearly seen to be coherent for all state spaces Θ.

To show that the log function is the only solution that covers all state spaces, Θ, we need only provide an example where for coherence the g–divergence has to be the log function.

Proof of Theorem. Assume that Θ contains at least two distinct points, say θ_1 and θ_2. Otherwise, π is degenerate and the thesis is trivially satisfied. To prove this theorem, it is sufficient to consider the case $n = 2$ and a very specific choice for π, taking $\pi = p_0\delta_{\theta_1} + (1-p_0)\delta_{\theta_2}$, where $0 < p_0 < 1$. Any probability measure ν absolutely continuous with respect to π has to be equal to $p\delta_{\theta_1} + (1-p)\delta_{\theta_2}$, for some $0 \leq p \leq 1$. Therefore, in this specific situation, the loss $L(\nu; I, \pi)$ becomes:

$$\begin{aligned} l(p, p_0, h_I) := {} & p\, h_I(\theta_1) \;+\; (1-p)\, h_I(y_1) \\ & + p_0\, g\left(\frac{p}{p_0}\right) \;+\; (1-p_0)\, g\left(\frac{1-p}{1-p_0}\right), \end{aligned}$$

where $h_I(\theta_i) = h(\theta_i, I_1) + h(\theta_i, I_2)$ for $I = (I_1, I_2)$ and $h_I(\theta_i) = h_1(\theta_i, I_j)$ for $I = I_j$, $i, j = 1, 2$. Denote by p_1 the probability $\pi_{I_1}(\{\theta_1\})$, i.e. the minimum point of $l(p, p_1, h_{(I_1,I_2)})$ as a function of p, and by p_2 the probability $\pi_{(I_1,I_2))}(\{\theta_1\})$. By hypotheses, p_2 is the unique minimum point of both loss functions $l(p, p_1, h_{I_2})$ and $l(p, p_0, h_{(I_1,I_2)})$. Again by hypothesis, we shall consider only those functions h_{I_1} and h_{I_2} such that each one of the functions $l(p, p_0, h_{I_1})$, $l(p, p_1, h_{I_2})$, and $l(p, p_0, h_{(I_1,I_2)})$, as a function of p, has a unique minimum point, which is p_1 for the first one and p_2 for the second and third one. The values p_1 and p_2 have to be strictly bigger than zero and strictly smaller than one: this was proved by Bissiri and Walker (2012) in their Lemma 2. Hence, p_1 has to be a stationary point of $l(p, p_0, h_{I_1})$ and p_2 of both the functions $l(p, p_1, h_{I_2})$ and $l(p, p_0, h_{(I_1,I_2)})$. Therefore,

$$g'\left(\frac{p_1}{p_0}\right) \;-\; g'\left(\frac{1-p_1}{1-p_0}\right) \;=\; h_{I_1}(y_1) \;-\; h_{I_1}(\theta_1), \tag{4}$$

$$g'\left(\frac{p_2}{p_0}\right) \;-\; g'\left(\frac{1-p_2}{1-p_0}\right) \;=\; h_{(I_1,I_2)}(y_1) \;-\; h_{(I_1,I_2)}(\theta_1), \tag{5}$$

$$g'\left(\frac{p_2}{p_1}\right) \;-\; g'\left(\frac{1-p_2}{1-p_1}\right) \;=\; h_{I_2}(y_1) \;-\; h_{I_2}(\theta_1). \tag{6}$$

Recall that $h_{(I_1,I_2)} = h_{I_2} + h_{I_1}$. Therefore, summing up term by term (4) and (6), and considering (5), one obtains:

$$\begin{aligned} & g'\left(\frac{p_2}{p_0}\right) \;-\; g'\left(\frac{1-p_2}{1-p_0}\right) \\ & \quad = g'\left(\frac{p_1}{p_0}\right) \;-\; g'\left(\frac{1-p_1}{1-p_0}\right) \;+\; g'\left(\frac{p_2}{p_1}\right) \;-\; g'\left(\frac{1-p_2}{1-p_1}\right). \end{aligned} \tag{7}$$

Recall that by hypothesis (4)–(6) need to hold for every two functions h_{I_1} and h_{I_2} arbitrarily chosen with the only requirement that p_1 and p_2 uniquely exist.

Hence, (7) needs to hold for every (p_0, p_1, p_2) in $(0,1)^3$. By substituting $t = p_0$, $x = p_1/p_0$ and $y = p_2/p_1$, (7) becomes

$$\begin{aligned} g'(xy) - g'\left(\frac{1-txy}{1-t}\right) \\ = g'(x) - g'\left(\frac{1-tx}{1-t}\right) + g'(y) - g'\left(\frac{1-txy}{1-tx}\right), \end{aligned} \tag{8}$$

which holds for every $0 < t < 1$, and every $x, y > 0$ such that $x < 1/t$ and $y < 1/(xt)$. Being g convex and differentiable, its derivative g' is continuous. Therefore, letting t go to zero, (8) implies that

$$g'(xy) = g'(x) + g'(y) - g'(1) \tag{9}$$

holds true for every $x, y > 0$. Define the function $\varphi(\cdot) = g'(\cdot) - g'(1)$. This function is continuous, being g' such, and by (9), $\varphi(xy) = \varphi(x) + \varphi(y)$ holds for every $x, y > 0$. Hence, $\varphi(\cdot)$ is $k \ln(\cdot)$ for some k, and therefore

$$g'(x) = k \ln(x) + g'(1), \tag{10}$$

where $k = (g'(2) - g'(1))/\ln(2)$. Being g convex, g' is not decreasing and therefore $k \geq 0$. If $k = 0$, then g' is constant, which is impossible, otherwise, for any h_I, p_1 satisfying (4) either would not exist or would not be unique. Therefore, k must be positive. Being $g(1) = 0$ by assumption, (10) implies that $g(x) = k\,x \ln(x) + (g'(1) - k)(x - 1)$. Hence,

$$h_2(\nu_1, \nu_2) = k \int \ln\left(\frac{\mathrm{d}\nu_1}{\mathrm{d}\nu_2}\right) \mathrm{d}\nu_1$$

holds true for some $k > 0$ and for every couple of measures (ν_1, ν_2) on Θ such that ν_1 is absolutely continuous with respect to ν_2.

Joint inference for quantiles and the Bayesian boxplot. We discuss this illustration for three reasons. The first is that there is a unique loss function for learning about a set of quantiles, countering the notion that loss functions are arbitrary, and second there is no traditional Bayesian version for updating a set of quantiles which can coincide with our approach. Finally, we show how boxplots, one of the most widely used exploratory graphical tool, can be enhanced by taking into account uncertainty in the plot due to a finite sample size.

Let us start with the median solely. The unique loss function for learning about the median of a distribution function is given by $l(\theta, x) = w|\theta - x|$ for some $w > 0$. Hence, the posterior distribution is given by

$$\pi(\theta|x_1, \ldots, x_n) \propto \exp\left\{-w\sum_{i=1}^{n}|x_i - \theta|\right\}\pi(\theta).$$

One might be tempted to argue that this is merely a Bayesian update using the Laplace distribution and hence falls within the Bayesian paradigm. This is correct but it would put the Bayesian in an awkward quandary if she knew, for example, the observations were coming from a normal distribution.

In fact we are, as we have stated previously, not assigning a probability model for x. To make this distinction more explicit let us consider the situation where we want to learn about the three quartiles $(\theta_1, \theta_2, \theta_3)$ jointly, where θ_1 is the lower quartile, θ_2 the median, and θ_3 the upper quartile. The prior will be denoted by $\pi(\theta_1, \theta_2, \theta_3)$ which would obviously include the constraint $\theta_1 < \theta_2 < \theta_3$. The loss function $l(\theta, x)$ in this case, treating the learning of the quartiles with equal importance, is given by

$$\begin{aligned} l(\theta, x) &= w\,\{0.25(\theta_1 - x)_+ + 0.75(x - \theta_1)_+ + \\ &\quad +0.5|\theta_2 - x| + 0.75(\theta_3 - x)_+ + 0.25(x - \theta_3)_+\} \end{aligned}$$

for some $w > 0$. Then the posterior distribution is given by

$$\pi(\theta|x_1, \ldots, x_n) \propto \pi(\theta)\exp\left\{\sum_{i=1}^{n} l(\theta, x_i)\right\}.$$

This can not be obtained by any Bayesian model that has currently been proposed. It is certainly therefore not classifiable as a Bayesian update.

We can illustrate the utility of this by considering a boxplot. In Fig (1) we show a boxplot of data taken from the example used in MATLAB help file for the function `boxplot.m`, in the statistics toolbox. The plot illustrates the distribution of miles per gallon (MPG) from records of a selection of cars taken in the 1970s, broken down by manufacturing country. The data set is available as

`carbig.mat` in MATLAB, we have omitted the "England" group which contains only 1 observation.

The boxplot is one of the most important and widely used graphical tool applied to summarise the distribution of data and highlight potential differences in the distributions across groups, but there is traditionally no uncertainty displayed in the summary statistics of the distributions used in the boxplot. In fact, for this data there are only 13 observations for "French" cars while there are 249 observations for the "USA", yet the conventional boxplot fails to inform on this.

We placed a prior on the median, upper and lower quartiles defined by the blue boxes in Fig (1) and account for the uncertainty by inferring the posterior distribution on these unknowns. Let θ_1 denote the lower quartile, θ_2 the median and θ_3 the upper quartile. We adopted a normal, fairly vague, prior, $\theta_1 \sim N(10, 100)$; $\theta_2 \sim N(20, 100)$; $\theta_3 \sim N(30, 100)$, with the constraint $\theta_1 < \theta_2 < \theta_3$. We adopt the "observed unit information loss" in the setting of w, see Section 3, so

$$\widehat{w} = \frac{\int \log\{\pi(\widehat{\theta})/\pi(\theta)\}\, \pi(\mathrm{d}\theta)}{\frac{1}{n-p}\sum_{i=1}^{n} l(\widehat{\theta}_x, x_i)},$$

where we estimate $\int \log\{\pi(\widehat{\theta})/\pi(\theta)\}\, \pi(\mathrm{d}\theta)$ via Monte Carlo methods and use a Nelder-Mead optimiser for $\widehat{\theta}_x$.

We then implemented a Metropolis-Hastings MCMC algorithm to sample from the posterior $\pi(\theta_1, \theta_2, \theta_3|x)$, for each of the 6 groups of cars shown in Fig (1), using 100,000 samples with a 50,000 sample burn-in.

In Fig (2) we show our "Bayesian boxplot" which includes the original boxes (empirical estimates) overlaid with 95% credible intervals for $(\theta_1, \theta_2, \theta_3)$. Credible intervals are shown as extended dotted lines from the empirical estimates with a small diamond denoting the edge of the interval. In comparison with Fig (1) we see that Fig (2) contains much more information. For example, we see that while in Fig (1) the median MPG of Italian and Swedish cars look different, in fact the 95% credible intervals overlap in Fig (2). In addition we see that there is considerable overlap in the distribution of medians between Sweden and the USA; and in general, comparison of medians or distributions in the conventional boxplot are obscured and confounded by sample size.

The MCMC samples approximately from $\pi(\theta_1, \theta_2, \theta_3|x)$ for France and USA are shown in Figs (3), (4). The data set for France contains 13 observations and hence there is much greater uncertainty in the posterior marginals. Moreover, looking at the joint densities of (θ_1, θ_2) and (θ_2, θ_3) we can see the constraints imposed by the prior. In contrast, due to the higher sample size the posterior samples for the USA are tighter and hence exhibit less dependence. An interesting extension would be to include hierarchical priors on the quartiles whereby one could borrow strength across groups.

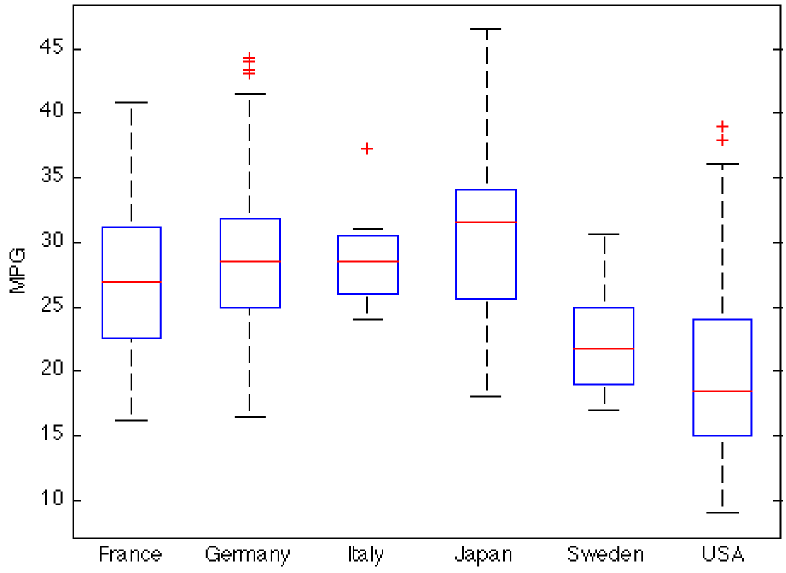

Figure 1: Boxplot of cars MPG data; taken from the MATLAB boxplot.m help file illustration.

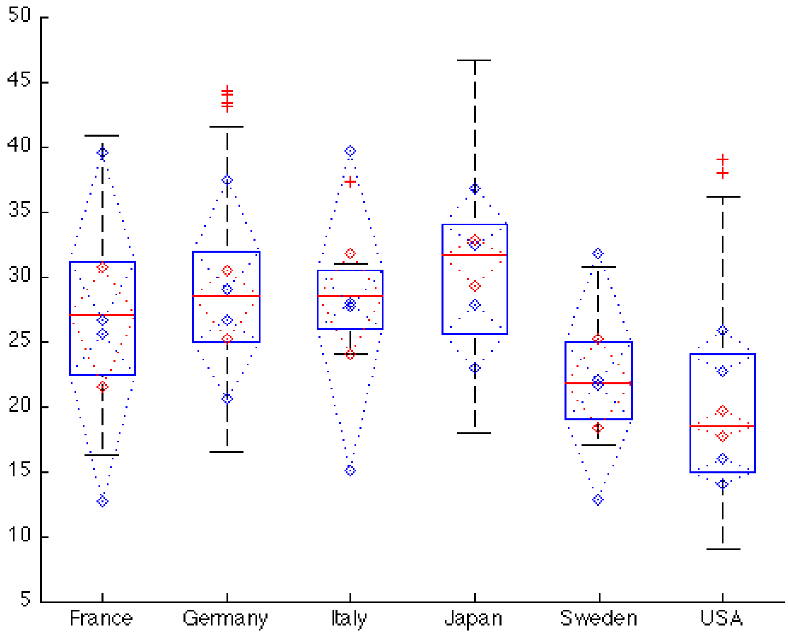

Figure 2: General Bayesian Boxplot of cars MPG data using Unit Information Loss

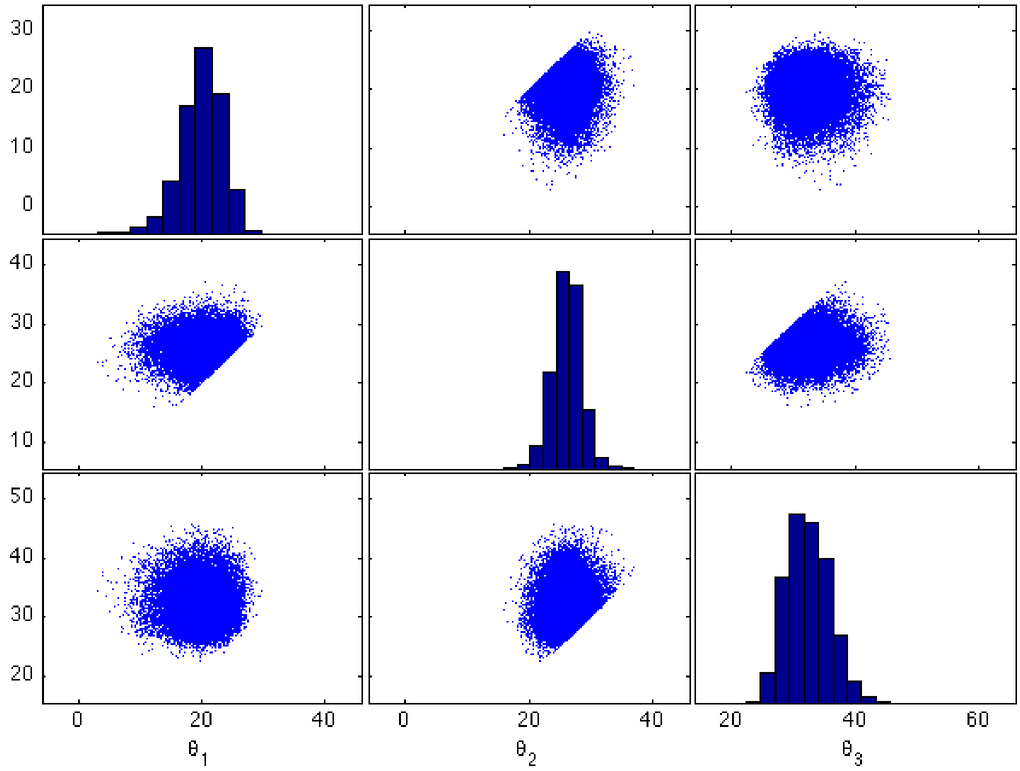

Figure 3: Posterior samples for quartiles of Franch cars MPG data using Unit Information Loss

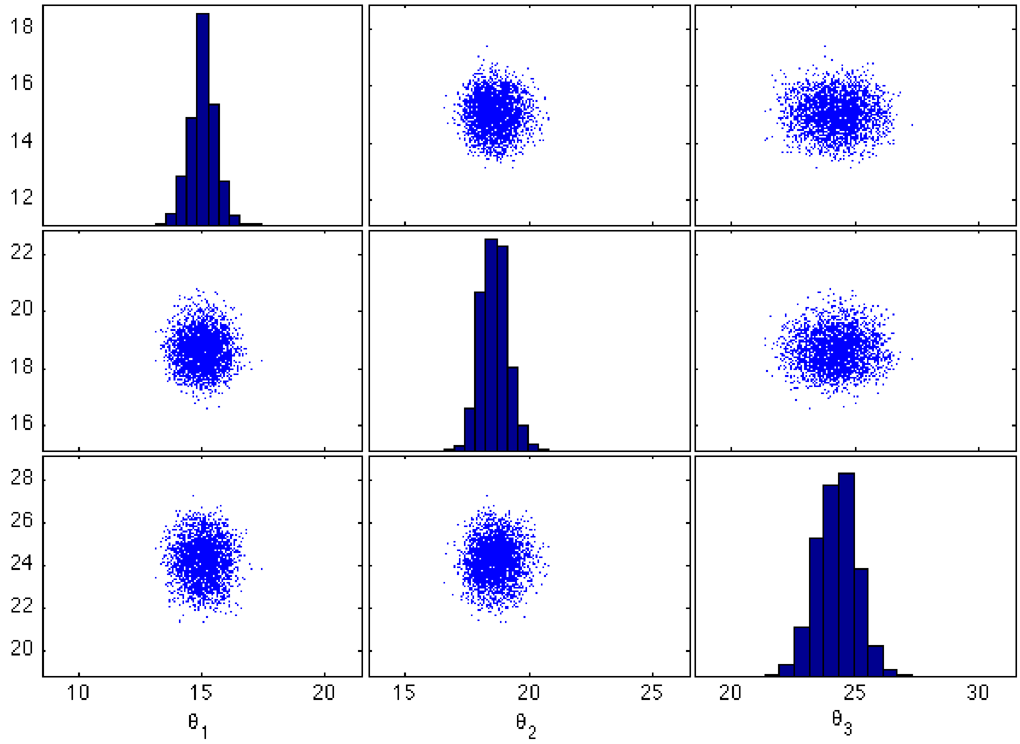

Figure 4: Posterior samples for quartiles of USA cars MPG data using Unit Information Loss
